# Morphology and paleoecology of a hybodontiform with serrated teeth, *Priohybodus arambourgi*, from the Late Jurassic of northeastern Brazil

**DOI:** 10.1002/ar.25671

**Published:** 2025-04-20

**Authors:** Estevan Eltink, Kelly Roberta da Silva, Marco Aurélio Gallo de França, Débora Melo Ferrer de Morais, Matías Soto, Christopher J. Duffin

**Affiliations:** ^1^ Colegiado de Ecologia Universidade Federal do Vale do São Francisco Senhor do Bonfim Bahia Brazil; ^2^ Programa de Pós‐Graduação em Ecologia e Evolução Universidade Estadual de Feira de Santana Feira de Santana Bahia Brazil; ^3^ Colegiado de Ciências Biológicas Universidade Federal do Vale do São Francisco Petrolina Pernambuco Brazil; ^4^ Serviço Geológico do Brasil—CPRM SUREG‐RE Recife Pernambuco Brazil; ^5^ Facultad de Ciencias Instituto de Ciencias Geológicas Montevideo Uruguay; ^6^ Earth Science Department The Natural History Museum London UK

**Keywords:** Hybodontiformes, Sharks, Elasmobranchii, Morphometrics, Aliança Formation, Tucano Basin

## Abstract

Hybodontiformes was a diverse, successful, and important group of shark‐like chondrichthyans known from a variety of ecosystems. Some representatives of the order had a wide palaeogeographic distribution, as is the case with *Priohybodus arambourgi*. With a multicuspidate crown, *P. arambourgi* was the first hybodontiform to develop fully serrated cutting edges on its teeth, a feature shared with many modern sharks (Neoselachii). Although Hybodontiformes comprises a group of early‐diverging sharks with relevant diversity and abundance in different ecosystems across the Paleozoic and Mesozoic, the morphometry of preserved teeth has been weakly explored. Here, we present the first record of this taxon for the Aliança Formation (Tithonian) of the Tucano Basin, northeastern Brazil. The results demonstrated morphometric correlations of the features encompassed in the central cusp and lateral cusplets, indicating a non‐allometric relationship in the increase of the crowns. *P. arambourgi* had a homodont dentition that preserves an ante‐mortem wear pattern on the top of the central cusp, an important trait recognized as result for prey preferences. A comparison of the assemblages of *P. arambourgi* also demonstrates intraspecific variation among populations in Gondwana, indicating morphological plasticity of this species from Africa and South America. Finally, we compare the morphology of *P. arambourgi* with modern sharks, inferring the likely size, feeding mechanisms and prey preferences for a hybodontiform.

## 1 INTRODUCTION

Representing most of the diversity among extant elasmobranchs Chondrichthyes, sharks and rays have a worldwide distribution, occupying freshwater and marine ecosystems (Ebert et al., 2021; Last et al., 2016; Weigmann, 2016). Many other extinct chondrichthyan orders, such as Cladoselachiformes, Symmoriiformes, Xenacanthiformes, Ctenacanthiformes, and Hybodontiformes, date back to the Middle Paleozoic, also showing a remarkable diversity (Friedman & Sallan, 2012; Schnetz et al., 2024). Among these orders, only the Hybodontiformes and Neoselachii survived to the end of the Mesozoic, while other groups were decimated during mass extinction events at the end of the Paleozoic or Mesozoic (Benton & Benton, 2015; Feichtinger et al., 2018; Guinot et al., 2013). In the Cenozoic, the only elasmobranch lineage that persisted was members of the Neoselachii (Maisey et al., 2004). Many Mesozoic and Cenozoic shark families known by their fossil record also have living representatives (Paillard et al., 2020; Pimiento & Benton, 2020). By comparison, aspects of their natural history that are not preserved in the geological record could be inferred as size, prey preference, and feeding mechanism (Cooper et al., 2023).

The Hybodontiformes share similarities with modern Neoselachii with which they may share exclusive common ancestry (Maisey, 2012). The group is characterized by shark‐like bodies with two dorsal fins supported by convex spines and cephalic hooks behind the orbit (in males) (Maisey, 1978, 1982; Maisey et al., 2004). The group temporally spans from the Mississippian (Carboniferous) to the Cretaceous, with individual species varying in size from a few centimeters to a few meters in length (Nelson, 2006; Nelson et al., 2016). The Hybodontiformes experienced a significant diversity in the Triassic, becoming abundant shark‐like elasmobranchs until the end of the Jurassic (Duffin & Thies, 1997; Leuzinger et al., 2017; Stumpf et al., 2022, 2023). Along the Mesozoic, other groups such as Elasmobranchii (Neoselachii) began to diversify (Guinot & Cavin, 2016; Kriwet et al., 2009; Sternes et al., 2024). The diversification was well established by the Tithonian, despite most modern orders appearing earlier in the Jurassic (Cappetta, 2012; Thies, 1981), probably forcing the Hybodontiformes to inhabit more restricted areas, such as freshwater ecosystems (Cuny et al., 2009; Rees & Underwood, 2008). In fact, isolated teeth and spines of Hybodontiformes are still found associated with freshwater deposits, contrasting with the dominance of modern sharks that inhabited marine ecosystems (Everhart, 2011; Fischer, 2013). The taxonomy of the Hybodontiformes has a significant complexity, showing some uncertainties for the proposition of families and systematic positions of taxa. Even so, during the Mesozoic, some families are traditionally recognized, such as Hybodontidae, Polyacrodontidae, Acrodontidae, and Lonchidiidae (Cappetta, 2012; Rees, 2008; Underwood & Rees, 2002).

The iconic genus *Hybodus* (Agassiz, 1833–1844) represents the family Hybodontidae—hybodontiforms that stand out due to their abundance in fluvial‐lacustrine and marine environments, and wide distribution across continents in the Mesozoic (Cappetta, 2012). The Hybodontidae also includes the genus *Priohybodus*, a monospecific taxon represented by *Priohybodus arambourgi* (Duffin, 2001). The *P. arambourgi* was grouped with *Secarodus* and *Planohybodus* genera within an unnamed hybodontid family (Rees, 2008) that later was informally proposed as “Priohybodontines” (Soto et al., 2012).

The species was first described by d'Erasmo (1960) based on fragmented and isolated tooth crowns from the Late Jurassic of Somalia, North Africa. Since then, new discoveries have expanded its geographical distribution. Other specimens have been reported from the Early Cretaceous of southern Tunisia (Tabaste, 1963) and the Late Jurassic Lugh series of Somalia (Cappetta, 1987). Goodwin et al. (1999) reported *P. arambourgi* based on a partially complete crown from probable Tithonian deposits from Ethiopia. Duffin (2001) redescribed *P. arambourgi* based on approximately 200 teeth (complete or fragmented) and one dorsal fin spine found in the Chicla Sandstone (Aptian to Albian, Early Cretaceous), northwestern Libya. The taxon has also been found in northern Yemen, in the Amran Formation (Upper Jurassic) (Duffin, 2001). Cuny et al. (2004) also described *P*. *arambourgi* from the Lower Cretaceous (Aptian) of Tunisia, present in the Douiret Formation. Teeth of *P. arambourgi* were found in Late Jurassic rocks of Uruguay (Tacuarembó Formation), representing the first record from South America (Perea et al., 2001; Soto et al., 2012) (Table 1).

**TABLE 1:** Records of *Priohybodus arambourgi* in Mesozoic of Gondwana.

Localities—formations	Ages	References
Somalia—Conca di El Mao	Late Jurassic	d'Erasmo (1960)
Somalia—Lugh Series of Gerdes el Abid	Late Jurassic	Cappetta (1987)
Ethiopia—Mugher Mudstone	Late Jurassic—Tithonian	Goodwin et al. (1999)
Tunisia—Douiret Formation	Early Cretaceous	Tabaste (1963); Cuny et al. (2004, 2010); Le Loeuff et al. (2010)
Libya—Chicla Sandstone	Early Cretaceous‐Aptian—Albian	Duffin (2001)
North Yemen—Amran Formation	Late Jurassic—Kimmeridgian—Tithonian	Duffin (2001)
Uruguay—Tacuarembó Formation	Late Jurassic	Perea et al. (2001); Soto et al. (2012)
Brazil—Aliança Formation (Tucano Basin)	Late Jurassic	This work

The Aliança Formation crops out in the Recôncavo‐Tucano‐Jatobá Rift System (RTJ). Most of the vertebrate remains were collected in the Jatobá Basin, which preserves a fossil fauna composed of hybodontiform sharks (*Planohybodus*), Actinistia (*Mawsonia*), Dipnoi, and actinopterygian fishes (e.g., Lepisosteiformes). Recently, new crocodylomorph remains and an isolated vertebra of a basal neotheropod dinosaur have been added to the known diversity of the Aliança Formation in the Jatobá Basin (Carvalho et al., 2021; De Oliveira et al., 2022). However, few paleontological studies regarding vertebrate fossils have been dedicated to the contents of the Tucano Basin.

Paleontological studies in the Aliança Formation from the Tucano Basin began in the early 20th century, with the recorded occurrence of the pteridophyte *Phlebopteris branneri* (van Konijnenburg‐ Cittert, 1993; White, 1913). At the end of the 1930s, paleontological studies carried out by de Melo Jr and de Oliveira (1939) and Price (Brasil, Ministério da Agricultura, 1941) resulted in the identification of many new fossiliferous localities in the Tucano Basin. These fossiliferous sites have been mapped since the first half of the 19th century, but few paleontological studies concerning the Aliança Formation have appeared since then. Recent fieldwork was carried out on the western margin of the Tucano Basin, resulting in the recovery of an interesting paleofauna, which includes teeth of hybodontiformes (*Priohybodus* and *Planohybodus*), teeth and dorsal osteoderms of crocodyliforms, scales and teeth of Lepisosteiformes, bone fragments of *Mawsonia*, and coprolites (Eltink et al., 2018).

We present here the first record of *P. arambourgi* in the Aliança Formation (Tucano Basin), based on hundreds of teeth. The new record expands the known geographical distribution of the genus throughout South America. Different statistical approaches employed in this paper provide a comparison gathered from other records of *P. arambourgi* in Gondwana, along with extant sharks (lamniformes) that have a similarity in tooth morphology. Understanding that some shark functional traits are correlated to tooth morphology (Ciampaglio et al., 2005; Cooper et al., 2023; Frazzetta, 1988), the tooth measurements observed in *P. arambourgi* were used here as proxies to infer the ecology of this Mesozoic hybodontiform from Gondwana.

## 2 MATERIAL AND METHODOLOGY

### 2.1 Geological context

The Tucano Basin is part of the RTJ, an intracontinental rift associated with the rupture of West Gondwana during the formation of the eastern Brazilian margin (Alvarez & Holz, 2022; Heine et al., 2013; Milani et al., 1988; Szatmari et al., 1985; Szatmari & Milani, 1999). Most of the sedimentary deposition of the RTJ occurred during the Cretaceous with the implantation of half‐grabens. However, the pre‐rift sequence, such as the Aliança and Sergi formations (Brotas Group), was deposited during the Upper Jurassic (Brito, 1987; Guimarães, 2002). This stratigraphic pre‐rift sequence is marked by the recurrence of a fluvial‐eolian cycle (Sergi formation) and lacustrine transgressions (Aliança Formation). Conglomerates and sandstones, in addition to reddish shales and siltstones, characterize the phase comprising the continental, shallow, and perennial lakes with fluvio‐deltaic influence of the Aliança Formation (Brito, 1987; Guzmán‐González et al., 2016; Viana et al., 1971). The Aliança Formation, together with formations of the eastern margin of Upper Jurassic sequences from Brazil, such as the Brejo Santo and Bananeiras formations, represents the most northerly occurrence of sediments associated with the Afro‐Brazilian Depression, with association to the Gabon, Congo, and Cabinda basins (Africa) (Da Rosa & Garcia, 2000; Netto & Oliveira, 1985; Souto & Fernandes, 2017). Biostratigraphically, the lacustrine ostracods and palynological records placed the Aliança Formation in the Tithonian (Almeida‐Lima et al., 2022; Da Rosa & Garcia, 2000; Guzmán‐González et al., 2016; Müller, 1966; Regali et al., 1974; Regali & Viana, 1989; Viana et al., 1971). Alternatively, ages based on radioisotope dating (Rb‐Sr) placed the Aliança Formation in the Early‐Late Jurassic (Thomaz Filho & Lima, 1981) or even in the Late Triassic (Silva et al., 2012).

**FIGURE 1:**
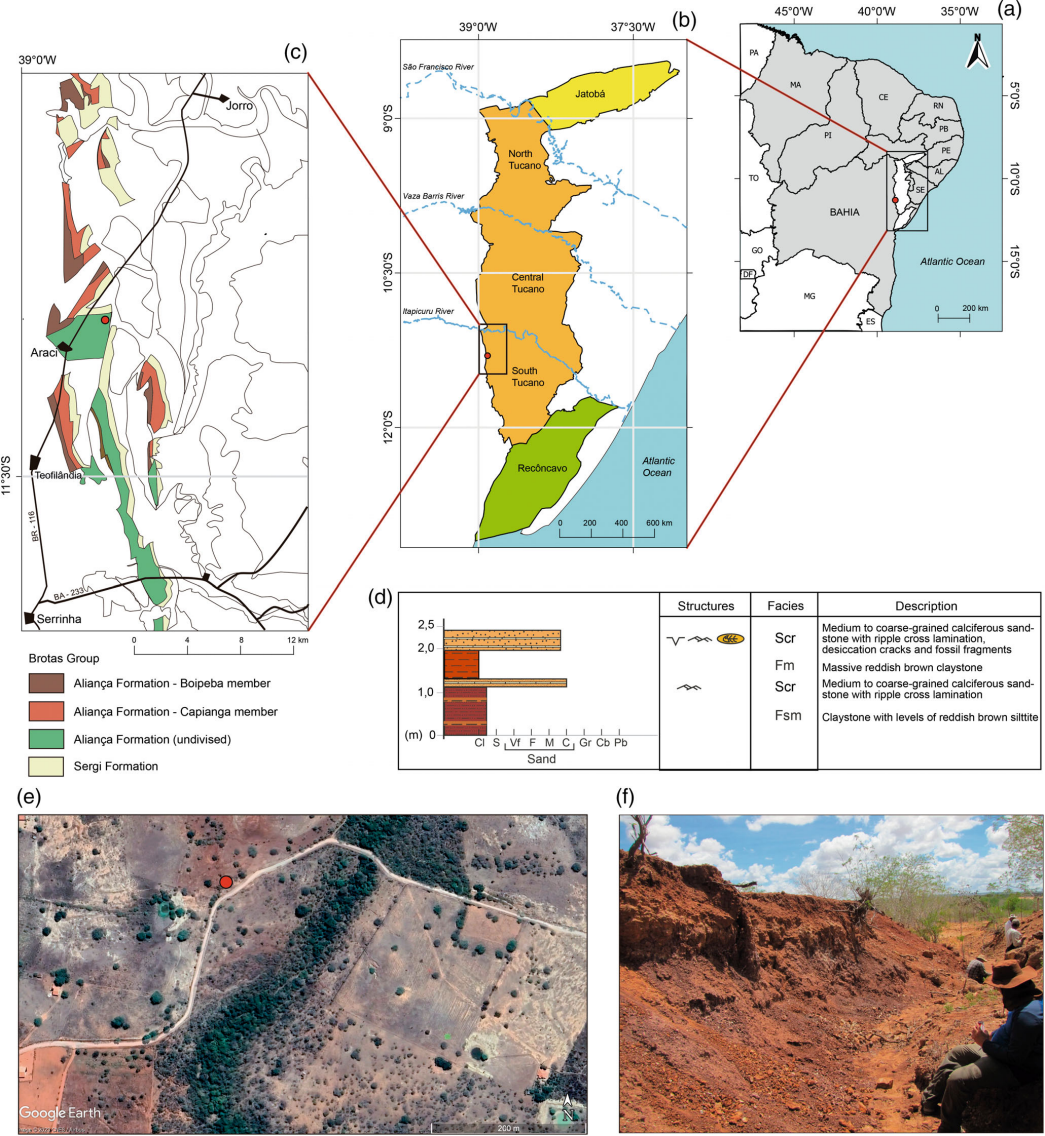
Geological settings. (a) System Recôncavo‐Tucano‐Jatobá (RTJ) in the context of northeastern Brazil. Red dots indicate the location of the outcrop. (b) Map of the RTJ basins, showing the Recôncavo (green), Jatobá (yellow), and Tucano (Orange) basins. The Tucano Basin is subdivided into North, Central, and South sub‐basins. (c) Geological map of the northwestern region of the South Tucano sub‐basin, showing in color the Brotas Group formations and members. Modified from Guimarães (2002). (d) Sedimentary log of the Aliança Formation outcrop, indicating the level of *Priohybodus arambourgi* (Src). (e, f) Satellite and general views of the outcrop, respectively.

The outcrop that yielded *P. arambourgi* is located in the western margin of the south sub‐basin of Tucano, in Araci County, Bahia, Brazil (Figure 1). The outcrop is characterized predominantly by mudstones of the Aliança Formation that were classified during geological mapping as an “undivided” Member of Aliança Formation, in this case, belonging neither to the Boipeba nor to the Capianga members (Guimarães, 2002). In general, the outcrop exhibits a low vertical exposure. In some portions, an intercalation between silty claystone with reddish brown color, and medium to coarse‐grained calciferous sandstones (Scr lithofacies) can be identified. The calciferous sandstone (Scr) has ripple cross‐lamination, bearing at the top of the outcrop the occurrence of desiccation cracks and fossil fragments (Figure 1e). The code proposed by Miall (1996) for the classification and interpretation of lithofacies has been adopted here. The fossils are associated with the Scr facies and found together with pebbles and claystone intraclasts (Figure 1e). The fossils have a fragmentary state of preservation, without any evidence of articulation.

The intercalation of massive reddish pelites with sandstone layers may represent lacustrine deposits in the area. The presence of ripple cross‐laminations suggests some influence of unidirectional tractive flow. The occurrence of fossil fragments of fishes and the reddish coloration of the rocks allow us to interpret that the facies association belongs to the Capianga Member. The area still exposes sandstones at the base of the ground with medium‐grained sandstones, reddish, either massive rocks or with incipient plane‐parallel stratification. Most likely, these sandstones originated in the fluvial environment of the Boipeba Member.

### 2.2 Analyses

The measurements and classifications gathered from the teeth of the Aliança Formation assemblage used 153 isolated teeth of *P. arambourgi*. The material is deposited in the palaeontological collection of the Federal University of the São Francisco Valley, Senhor do Bonfim, Bahia, Brazil, under the acronym LAPAL/SBF. Morphological terminology mostly follows Cappetta (2012).

**FIGURE 2:**
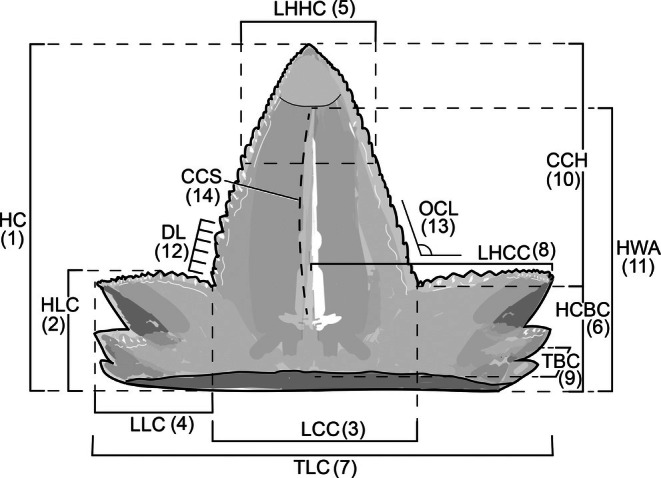
Measurements of the tooth crown used in this study based on *Priohybodus arambourg*i from the Aliança Formation, Tucano Basin, Brazil: HC—height of tooth crown (1); HLC—height of lateral cusplets (2); LCC—mesiodistal length of the central cusp at the base (3); LLC—mesiodistal length of lateral cusplets (4); LHHC—mesiodistal length at the half‐height of the central cusp (5); HCBC—height of the crown base at central cusp (6); TLC—total mesiodistal crown length (7); LHCC—distance from the most distal point of the lateral cusplets until the half‐length of the central cusp (8); TBC—thickness at the base of the crown (9); CCH—height of the central cusp (10); HWA—distance measured from the ante‐mortem wear facet to the crown base (11); DL—length of a single denticle (12); OCL—orientation (inclination) of the lateral cusplets relative to the central cusp (<45°—0; 45° to 90°—1) (13)*; CCS—curvature of lingual surface in the central cusp (absent—0, weak—1, or well‐marked—2) (14)*. (*) qualitative characters.

The selection of the morphological features for analytical assessment resulted in 14 variables, among which 12 variables are quantitative and 2 variables are qualitative characters (Figure 2). Among the quantitative characters, the selection includes the mesiodistal length and height of the central cusp, and lateral cusplets, characters commonly used in morphometric analyses of shark teeth (e.g., Kriwet et al., 2015; Marramà & Kriwet, 2017). The newly proposed quantitative characters include the labiolingual thickness of the teeth, the area of ante‐mortem wear at the tip of the central cusp, and the length of denticles. A single denticle length was taken from the average obtained from 5 mL at the base of the cutting edge in the central cusp. When both sides of the central cusp were present, the calculation included an average of both sides. The same applied to the lateral cusplets from both mesial and distal sides. All measurements were taken in millimeters. For incomplete teeth, the variables lacking information were treated as missing data.

The qualitative characters include the orientation (inclination) of the lateral cusplets relative to the central cusp and the curvature of the lingual surface on the central cusp, observed in lateral view (Figure 2: characters 13 and 14). These were used as grouping characters in the multivariate analyses (principal component analysis [PCA]). All the analyses employed the program Paleontological Statistics Software Package for Education and Data Analysis (PAST), version 4.16 (Hammer et al., 2001).

We adopted three different approaches to the analyses of the morphology in *P. arambourgi*. In the first, we observed the variation among the characters within the tooth assemblage of the Aliança Formation. This was based on PCA, a multivariate ordination commonly used for the investigation of biological variation (Sanguansat, 2012). For this purpose, as a starting point, all the characters were initially log‐transformed for normality and subsequently had a selection for the final comparison in the PCA. The first step of selection encompassed a Spearman's Rank correlation for the exclusion of highly correlated characters. Characters with high statistical correlation (more than 90%) were excluded, resulting in the exclusion of characters 5 (LHHC) and 10 (CCH), which were strongly correlated with character 1 (HC). The second step comprised a posteriori selection, in which the exclusion aimed at overlaid vectors after successive PCA analyses, removing noise and redundancy of the data (Kassambara, 2017). For the analysis of the data in the PCA, we considered a correlation matrix as character 11 (HWA) is non‐parametric. The vectors relating to characters 4 (LLC) and 8 (LHCC) were highly congruent with the vector corresponding to character HLC (2). In this case, the height of the lateral cusplets (HLC) was strongly correlated with the mesiodistal length of the lateral cusplets (LLC) and with the distance from the most distal point of the lateral cusplets until the half‐length of the central cusp (LHCC). The results of the final selection of characters are shown in Figure 4.

In *P. arambourgi*, a high triangular central cusp with serrated edges is similar to the condition of some extant sharks (Duffin, 2001). However, the rarity among Hybodontiforms with serrated cutting edges on the teeth is evident in comparison to the living sharks. An explanation seems to be the primitiveness of the enameloid microstructure in Hybodontiforms (Cuny et al., 2018). We observe a worn area at the top of the cusps that varies according to the size characters of the central cusps and lateral cusplets. So, the objective of this first approach was to assess how this feature is associated with others in the *P. arambourgi*. Additionally, as Duffin (2001) postulated that gentle teeth asymmetry observed in *P. arambourgi* does not modify their general morphology and may be associated with the pattern of monognathic heterodonty, we investigated this qualitative character, testing whether the slight asymmetry could also be associated with some feature.

The teeth of *P. arambourgi* are morphologically well uniform even bearing different occurrences of the species, which could result in some variation (Cuny et al., 2004; Duffin, 2001; Goodwin et al., 1999; Perea et al., 2001; Soto et al., 2012). In the second approach, we compare different tooth assemblages of *P. arambourgi*. We aimed to identify some dental variation in different assemblages recorded in Gondwana. In this context, tooth assemblages from the Chicla Formation (Libya) and the Tacuarembó Formation (Uruguay) were analyzed for comparative purposes, using measurements taken from 74 and 72 isolated teeth, respectively. All of the material was measured firsthand by the authors. The characters used encompass the total length, height, thickness, and denticle length on teeth from the various localities (Figure 2—1, 7, 9, 12). Firstly, selected morphometric variables were subjected to a multivariate analysis of variance (MANOVA) using a significance level of *p* = 0.05, with Bonferroni correction. The data were log‐transformed and tested with Shapiro–Wilk (0.05 level of significance) for normalization. Afterwards, we employed a Tukey's pairwise test to check if features differ significantly from each other in a post‐hoc pairwise comparison, describing specifically the differences of the variance among features for each locality. For a visualization in the morphospace of the effect of denticles among the assemblages, we employed a multivariate ordination in the PCA under variance–covariance matrix. Secondly, to test if denticle density decreases as the tooth gets larger in *P. arambourgi* (see Cuny et al., 2004; Perea et al., 2001), a regression analysis was applied to the height, length, and thickness of the teeth, comparing with denticle size from different assemblages of the species.

**FIGURE 3:**
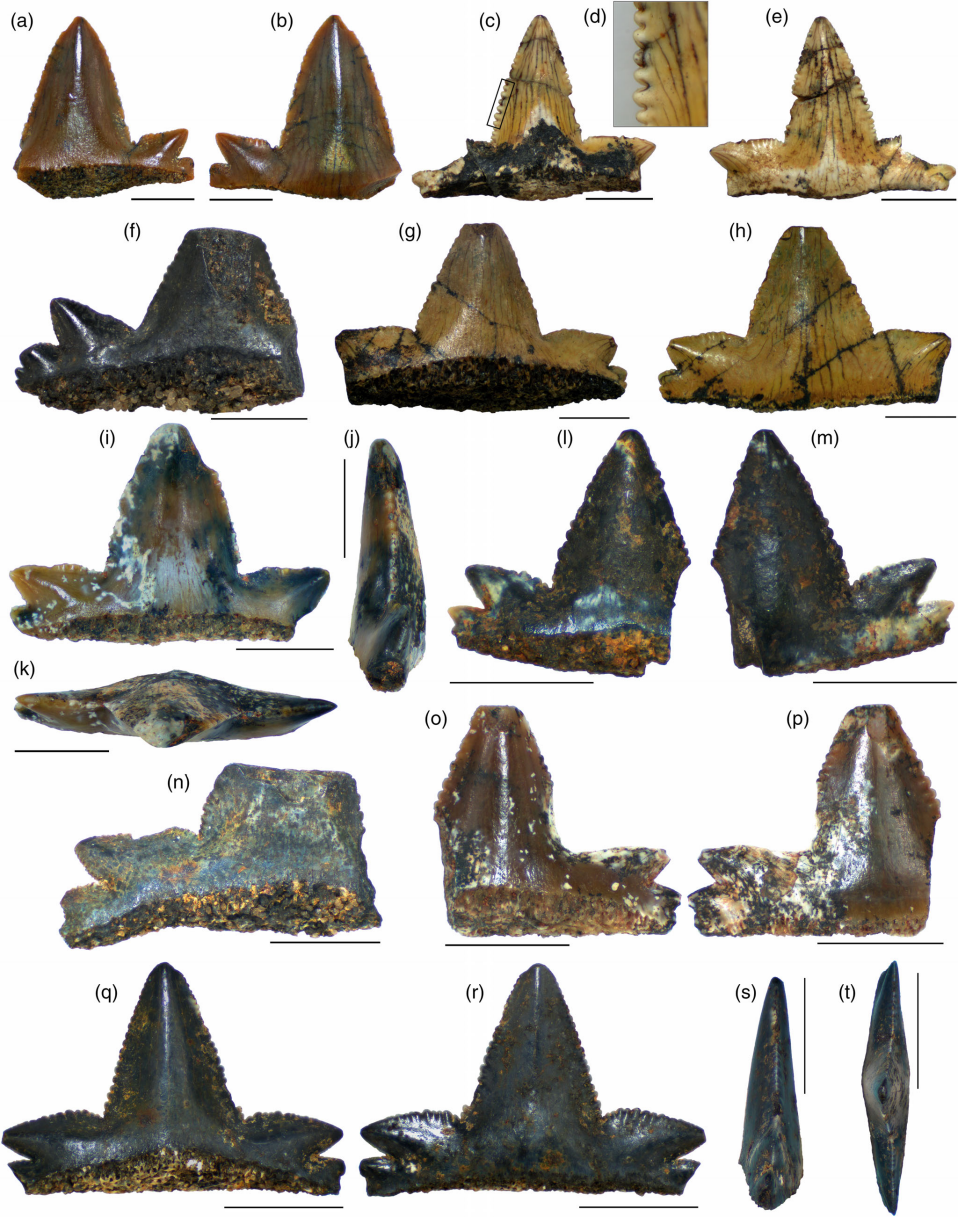
*Priohybodus arambourgi* from the Aliança Formation of Brazil (Upper Jurassic). (a and b) LAPAL/SBF‐2‐001 in lingual and labial views, respectively; (c–e) LAPAL/SBF‐2‐002 in lingual (c) and labial views (e), with detail of the tooth serrations (d); (f) LAPAL/SBF‐2‐004 in lingual view; (g–h) LAPAL/SBF‐2‐005 in lingual and labial views, respectively; (i–k) LAPAL/SBF‐2‐006 in lingual, lateral, and occlusal views, respectively; (l) and (m) LAPAL/SBF‐2‐018 in lingual and labial views, respectively; (n) LAPAL/SBF‐2‐020 in lingual view; (o and p) LAPAL/SBF‐2‐022 in lingual and labial views, respectively; (q–t) LAPAL/SBF‐2‐127 in lingual, labial, lateral, and occlusal views, respectively. Scale: 5 mm.

**FIGURE 4:**
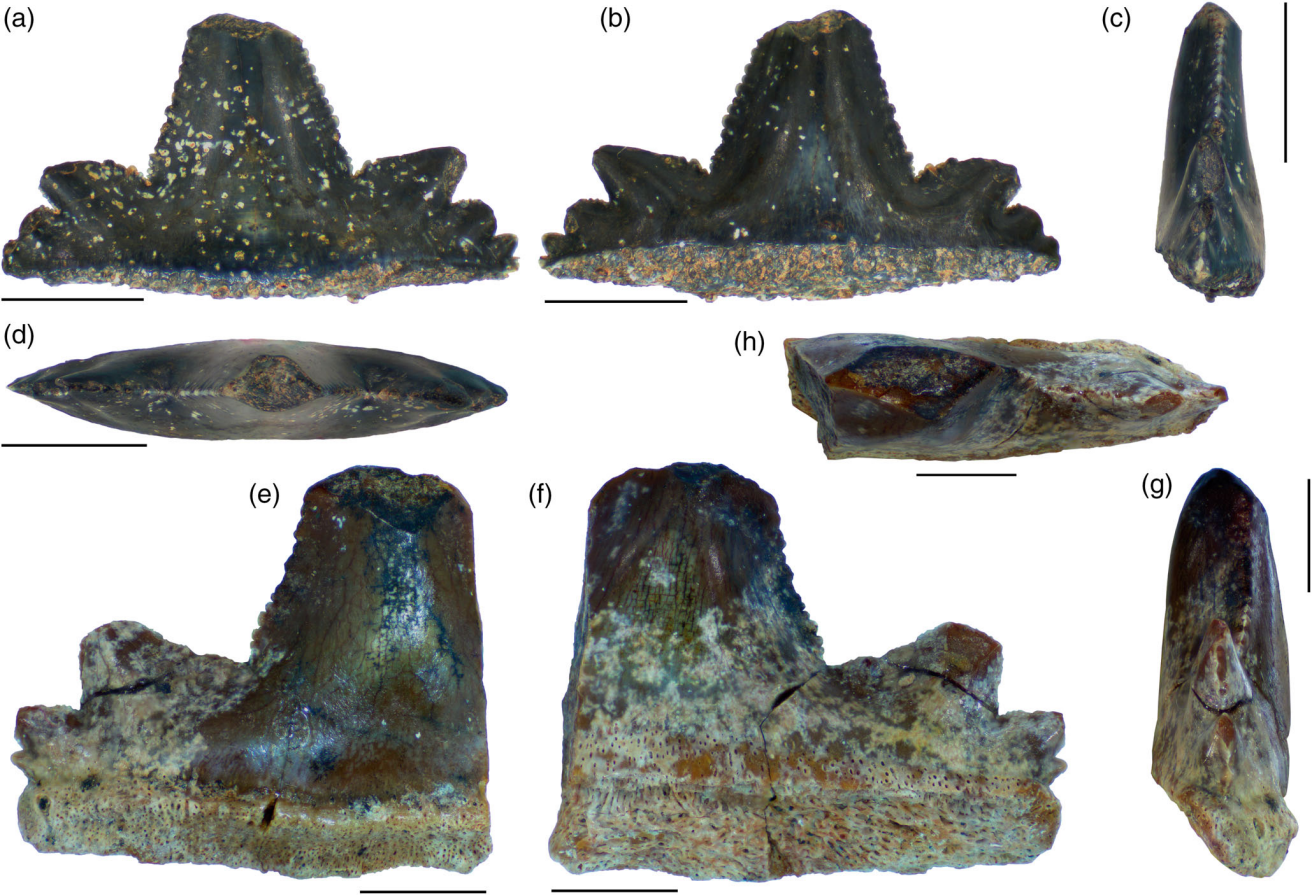
*Priohybodus arambourgi* from the Aliança Formation of Brazil (Upper Jurassic). (a–d) LAPAL/SBF‐2‐0164 in labial, lingual, lateral, and occlusal views, respectively; (e–h) LAPAL/SBF‐2‐0186 in labial, lingual, lateral, and occlusal views, respectively. Scale: 5 mm.

**FIGURE 5:**
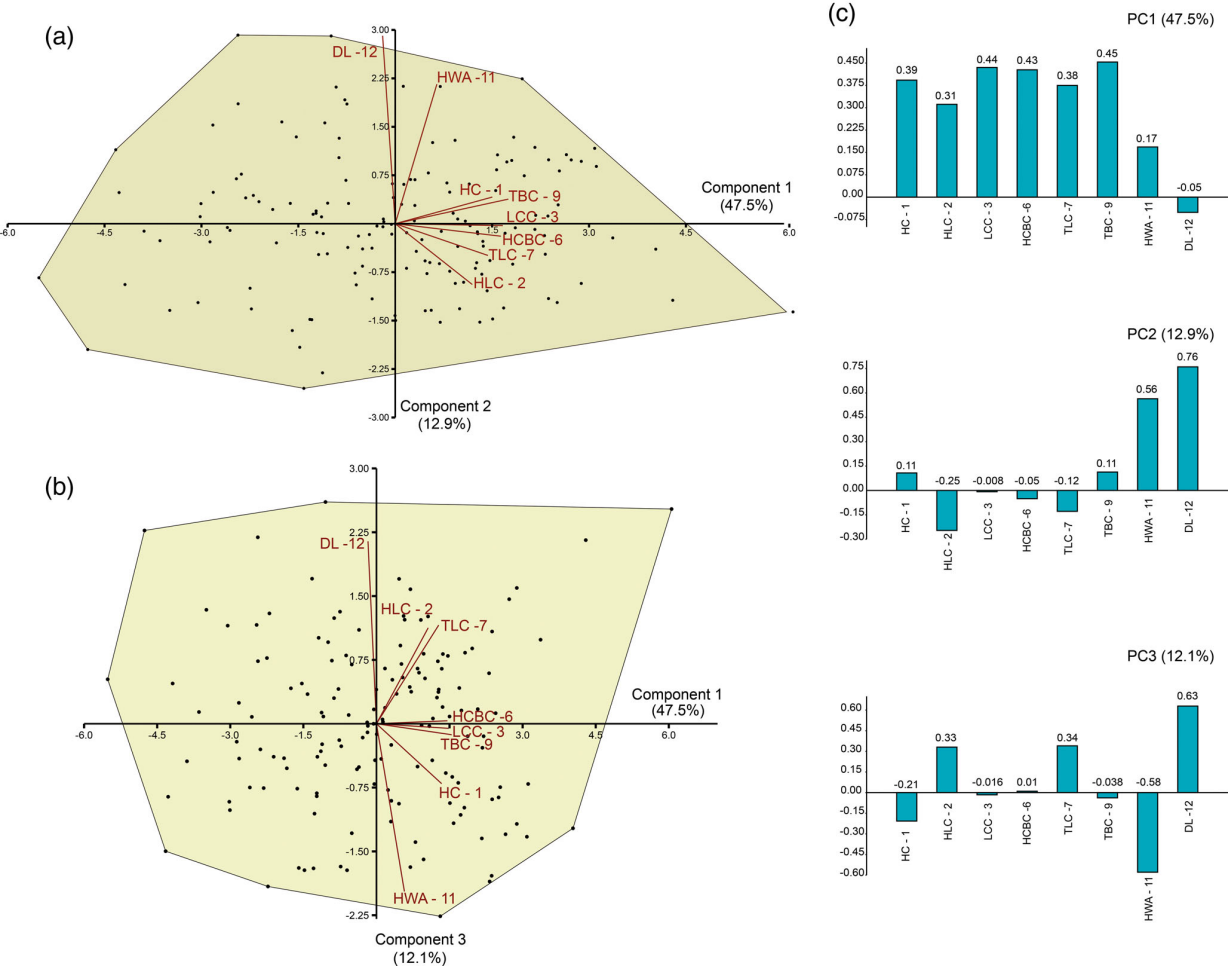
Results of the principal component analysis (PCA) performed on the log‐transformed measurements represented as convex hulls for *Priohybodus arambourg*i assemblage from the Aliança Formation; (a) Morphospace plotted on PC1 and PC2; (b) Morphospace plotted on PC1 and PC3; (c) loading values showing the variables associated with the first three principal component axes. For measurements abbreviation see Figure 2.

In the third approach, we made comparisons with a typical neoselachian lineage that bears a similar tooth morphology. Employing the database of Marramà and Kriwet (2017), we compared *P. arambourgi* with extant and fossil lamniformes. For this purpose, we used characters that encompass the observed variation in the size of the central cusp and lateral cusplets of *P. arambourgi* from the Aliança Formation assemblage (Figure 2—1–5, 7, 10). For this purpose, we also applied a multivariate ordination in the PCA with log‐transformed data comparing with the variance–covariance matrix. Finally, to infer paleoecological aspects based on tooth morphometry and morphology, we employ the correspondence of *P. arambourgi* with ecomorphotypes of extant sharks. We applied the classification of Cooper et al. (2023) for body size, feeding mechanism, and prey preference, understanding that some shark functional traits could be correlated with tooth morphology (Ciampaglio et al., 2005; Frazzetta, 1988).

## 3 RESULTS

### 3.1 Systematic paleontology

Chondrichthyes Huxley, 1880.

Elasmobranchii Bonaparte, 1838.

Hybodontiformes Maisey, 1987.

Hybodontidae Owen, 1846.

*Priohybodus* d'Erasmo, 1960.

*P. arambourgi* d'Erasmo, 1960.

### 3.2 Horizon and locality

The material of *P. arambourgi* was recovered from the Aliança Formation, in Upper Jurassic strata of the Tucano Basin. The fossil locality is addressed in the Araci municipality, Bahia, Brazil.

### 3.3 Material

LAPAL/SBF‐2‐001 to LAPAL/SBF‐2‐076, LAPAL/SBF‐2‐099 to LAPAL/SBF‐2‐111, LAPAL/SBF‐2‐117, LAPAL/SBF‐2‐123 to LAPAL/SBF‐2‐125, LAPAL/SBF‐2‐127 to LAPAL/SBF‐2‐142, LAPAL/SBF‐2‐147 to LAPAL/SBF‐2‐150, LAPAL/SBF‐2‐155 to LAPAL/SBF‐2‐184, LAPAL/SBF‐2‐186 to LAPAL/SBF‐2‐190, LAPAL/SBF‐2‐201 to LAPAL/SBF‐2‐205.

Institutional abbreviations: FC‐DPV, Colección de Vertebrados Fósiles, Facultad de Ciencias, Montevideo, Uruguay; LAPAL/SBF, Laboratório de Paleontologia, Universidade Federal do Vale do São Francisco, Senhor do Bonfim, Bahia, Brazil; NHMUK, The Natural History Museum, London, United Kingdom.

### 3.4 Description of the teeth

The material referred to this taxon from the Aliança Formation demonstrates a fragmentary state of preservation without any sign of articulation. In general, the teeth preserve the crowns without the root (except in a single example). No dorsal fin spines are associated with the material. The sample size is almost 200 teeth, whose completeness varies considerably, with most of the teeth preserving only the central cusp; some teeth are well‐preserved, with central cusp and lateral cusplets at the base. Some of the teeth exhibit marks of abrasion, rendering detailed observation of the details of the tooth surface difficult. However, in most of the teeth, a well‐preserved surface permits observation of the enameloid and denticles.

The Aliança Formation assemblage, and those from different localities of Gondwana, exhibits teeth morphology consisting of a multicuspid crown, bearing a wide central cusp that is triangular and compressed labiolingually. The central cusp is flanked mesially and distally by smaller lateral cusplets. The thickness of the central cusp does not vary by much along a single tooth. The axes of the lateral cusplets and the central cusp converge toward the base of the teeth. In addition, the central cusp has a gentle convex surface on the lingual side, while the labial region is distinguished by a flat surface. The central cusps have, in general, a smooth surface on both lingual and labial sides. Ornamentation of the crown is absent in the material described herein, differing from that observed in some large *P. arambourgi* specimens from Tunisia, which show the presence of irregular ridges (Cuny et al., 2004). The majority of the teeth from the Aliança Formation have a symmetrical shape. However, in some teeth, the central cusps have a slight distal inclination, as also seen in the Tacuarembó specimens (Perea et al., 2001; Soto et al., 2012).

In the Aliança Formation specimens, the apical region of the central cusp shows a remarkable wear surface. This feature is not commonly noticed in other *P. arambourgi* teeth. The wear surface occurs on both lingual and labial surfaces of the central cusp and shows no sign of enameloid regeneration. The outline of the wear surface is semicircular, with an apically orientated concavity. Some teeth exhibit a wear surface extending from the apical tip of the cusp to a point halfway down the height of the central cusp (Figure 3f,n), while in some of the cusps, the worn area covers a smaller portion of the cusp tip (Figures  3g,o,p and 4 a,e). This feature may be associated with the feeding mechanism, by tooth‐to‐tooth contact between opposing upper and lower teeth during the biting action of the jaws and the hardness and resilience of prey items (see Sections 3 and 4).

Denticles are visible along both mesial and distal cutting edges of the teeth. Each denticle has a rounded crest. Denticle size varies in a nearly homogeneous way, although irregular size changes are evident, especially on the central cusps (Figure 3c,d), as seen in specimens described by Duffin (2001), Perea et al. (2001) and Cuny et al. (2004). Nonetheless, the denticles of the specimens from Libya have a more square‐shaped outline, with smooth divisions in some of the denticles. According to Cuny et al. (2004) and Perea et al. (2001), denticle size and density vary according to the size of the tooth, and small teeth may concentrate more denticles per millimeter than larger ones (see the results below for this hypothesis).

Lateral cusplet height is about 30% or less than that of the central cusp (Duffin, 2001; Perea et al., 2001). The teeth from the Aliança Formation commonly present three observable pairs of lateral cusplets, but a fourth incipient pair is sometimes present (Figure 4b). This resembles the condition of four or five pairs of lateral cusplets seen in the teeth from the Tacuarembó Formation (Soto et al., 2012), but differs from the condition observed in the specimens from Tunisia in which the third pair of lateral cusps is rarely seen (Cuny et al., 2004). The first pair of lateral cusplets is relatively larger than the more mesial and distal ones (Soto et al., 2012). The lateral cusplets are morphologically different from the central cusp and their orientation varies from more inclined sidewards (mesially and distally), reaching 90° in relation to the main axis of the central cusp (Figure 3g–i,o–r), or being more upright closer to the central cusp, with a lesser inclination of 45° (Figure 3a,b,f). Similar variation in *P. arambourgi* is observed in the Libyan and Uruguayan specimens but differs from the isolated tooth from Tunisia that exhibits more erect second cusplets. In occlusal view, the tips of the lateral cusplets are aligned in the same plane as the central cusp, resulting in a straight line for the cutting edge (Figures  3s,t and 4c,d). However, the central cusp and lateral cusplets are occasionally inclined divergently, resulting in cusp apices that are directed in different planes compared to each other (Figures 3j and 4g,h).

Specimen LAPAL/SBF‐2‐0186 is the only tooth to preserve a fragmentary root (Figure 4e–h). Other preserved roots include only one specimen each from Libya (P65461), Uruguay (FC‐DPV 1609) and Tunisia (JBNS 1) (Cuny et al., 2004; Duffin, 2001; Soto et al., 2012). Regarding the absence of roots in most teeth, Böttcher (2024) considers root resorption during replacement as a typical feature of hybodontiforms. In general, the roots of *P. arambourgi* teeth are square‐shaped in lateral view and rectangular in labial view, being slightly projected lingually from the crown underside. In lateral view, the root seen in specimen LAPAL/SBF‐2‐0186 is more triangular basally, lacking part of the lingual projection (Figure 4g). Labially, the root shows a smooth longitudinal ridge disposed along the base of the crown length, lacking an enameloid surface. Basal to the ridge, a groove is formed with the same disposition. The basal margin of the root is parallel to the base of the crown, bearing a rectilinear outline. It differs from the apicobasal concavity observed in the basal margin in the Libyan specimen (Duffin, 2001). The root in the Aliança Formation specimen is proportionally lower with respect to crown height compared with other specimens. The surface of the root is perforated by numerous tiny and randomly distributed vascular foraminae. The foraminae can be clearly observed in the lingual face, being oriented apicobasally (Figure 4f). The foraminae form a weak and horizontal row that can be observed below the crown base along the length of the root. An irregular row of foraminae below the crown base is present on both sides of the specimens from Tunisia and Libya, repeating the anaulacorhize arrangement previously seen in *P. arambourgi* (Cuny et al., 2004; Duffin, 2001).

### 3.5 Tooth measurement data for the Aliança Formation assemblage

The PCA of the 153 teeth of *P. arambourgi* retrieved eight principal component axes, in which the first three components represent more than 72% of the variability. The morphospaces are plotted on the first three axes (Figure 5a,b).

The PC1 has 47.5% of the representation of variation in the sample. In the description of more representative variables, this component is particularly closely related to size measurements of the teeth, being represented in the *X* axis of Figure 5. The positive values in the loadings of the PC1 vary from 0.3 to 0.45, demonstrating that relevance in the variation of the measurements is mostly related to the heights of the crown (HC) and the lateral cusplets (HLC), the mesiodistal lengths of the crown (TCL) and the central cusp base (LCC), the height of the crown base (HCBC), and the thickness at the crown base (TBC). The correlation statistics of these measurements were also observed in Spearman's Rank correlation (Table 2), showing that *P. arambourgi* teeth increases proportionally in size, especially observing variables such as height, length, and thickness. The values related to the wear area height (HWA) and denticle length (DL) have low or even negative loading values (Figure 5c—PC1). The second relevant component (PC2) explains 12.9% of the variation in the sample, reaching loading values mostly related to the HWA and DL, which range from 0.56 to 0.76, respectively. The Spearman's Rank correlation (Table 2) demonstrates a weak correlation of HWA with other measurements, such as HC, LCC, and TBC. The DL is non‐correlated with any other measurement. This demonstrates that denticle measurement comprises a variable that may be interpreted as independent of size‐increasing characters in the tooth morphology of *P. arambourgi*.

**TABLE 2:** Spearman's Rank correlation among measurements, showing in the upper diagonal the *p*‐values with Bonferroni correction, and the lower diagonal the values for relative statistical correlation.

	HC—1	HLC—2	LCC—3	HCBC—6	TLC—7	TBC—9	HWA—11	DL—12
HC—1		2.23E−02	6.66E−16	4.05E−13	2,26E−06	9.34E−26	0.0056002	1
HLC—2	0.73861		1.12E−06	2.32E−07	1.72E−06	3.68E−08	1	1
LCC—3	0.80939	0.72124		4.26E−12	6.80E−08	2.31E−28	0.037031	1
HCBC—6	0.77984	0.70294	0.66496		9.62E−06	6.06E−18	1	1
TLC—7	0.76234	0.71325	0.68538	0.64237		3.78E−10	1	1
TBC—9	0.89545	0.74565	0.83395	0.74918	0.73985		0.00080971	1
HWA—11	0.39506	0.18796	0.29847	0.18044	0.22404	0.38099		1
DL—12	−0.20107	−0.10612	−0.10683	−0.11393	0.025618	−0.04203	0.0065062	

Abbreviations: DL, length of a single denticle; HC, height of tooth crown; HCBC, height of the crown base at central cusp; HLC, height of lateral cusplets; HWA, distance measured from the antemortem wear facet to the crown base; LCC, mesiodistal length of the central cusp at the base; TBC, thickness at the base of the crown; TLC, total mesiodistal crown length.

In the third component (12.1%), the morphospace plotted on PC1 and PC3 (Figure 5b) corroborates the relevance of the DL as a variable with the highest loading value (0.63). The variables related to the HCL and the TLC represent, respectively, loading values of 0.33 and 0.34, being related to the base of the crown teeth. Variables such as height (HCBC) and length (LCC) of the central cusp and TBC show an approximation to the results demonstrated by component 1. In this case, such variables indicate that the morphometry of the crown base is an important character. It is noteworthy that the negative loading value of the distance of the HWA, with −0.58, is diametrically opposed to the variable of the denticle length. But it contrasts with the biplot observed in PC2, representing an increase in the wear area of the central cusp accompanied by a related increase in denticle length. The correlation of the characters is not significant (Table 2), indicating that the measurement of neither character corresponds with characters related to the size of the crown.

**FIGURE 6:**
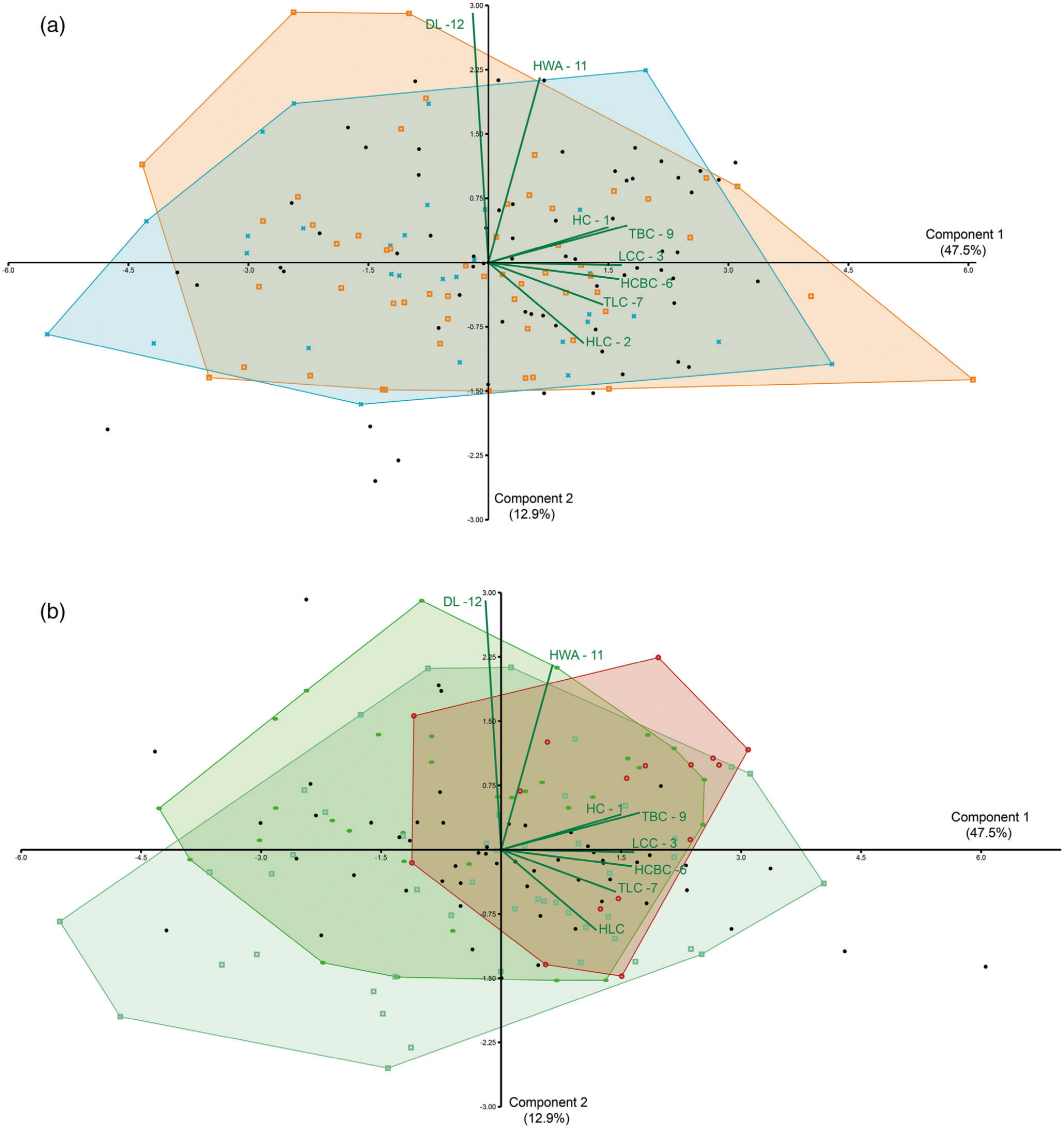
Morphospaces represented as convex hulls for the assemblage of isolated teeth of *Priohybodus arambourgi* from the Aliança Formation plotted on PC1 and PC2 grouped by qualitative characters. (a) Orientation of the lateral cusplets relative to the central cusps, in degrees. Squared points represent teeth with <45° (orange), “X” points represent teeth with 45° < 90° (blue). (b) Presence of curvature on lingual margin of the central cusp. Squared points represent the absence of curvature (light green), oval points represent teeth with weak curvature (green), circles represent teeth with well‐marked curvature (reddish brown). Teeth without information concerning qualitative characters selection are represented by black dots in both graphs.

Grouping the teeth using the presence of an acute orientation of the lateral cusplets relative to the central cusp (OLC) and the curvature of the lingual surface of the central cusp (CCS) results in a comparison in the morphospace in the PCA of qualitative characters, testing whether or not some variation in the observed tooth assemblage might be associated with other qualitative characters in the teeth (Figure 6). The results for the lateral cusplet inclination demonstrate significant congruency in the overlapping of morphospaces occupied by more inclined (blue) or lesser inclined (orange) lateral cusplet groups. Considering the variables in the analyzed assemblage, inclined lateral cusplets have the same attributes compared with other characters. The results observed concerning the presence of curvature on the lingual surface of the central cusps, and their degrees of curving, demonstrate a strong congruency between the group classifications (Figure 6b). The broader morphospace is related to teeth in which there is an absence of curvature (light green). The morphospace of the group with weak curvature in the central cusp shows an intermediate distribution, being followed by a more limited group with accentuated curvature that overlaps both previously mentioned groups. A comparison among the groups demonstrates that teeth with well‐marked curvature in the central cusp are concentrated in the morphospace related to larger teeth, considering the height, thickness, and length values on the PC1. Thus, this qualitative trait must be associated with the development of the teeth, being characteristic of teeth at a more advanced stage in ontogenetic development. As observed for the inclination of lateral cusplets, there is no qualitative variable here explored that is clearly associated with some perceptible heterodonty.

### 3.6 Comparisons among *P. arambourgi* tooth assemblages

**FIGURE 7:**
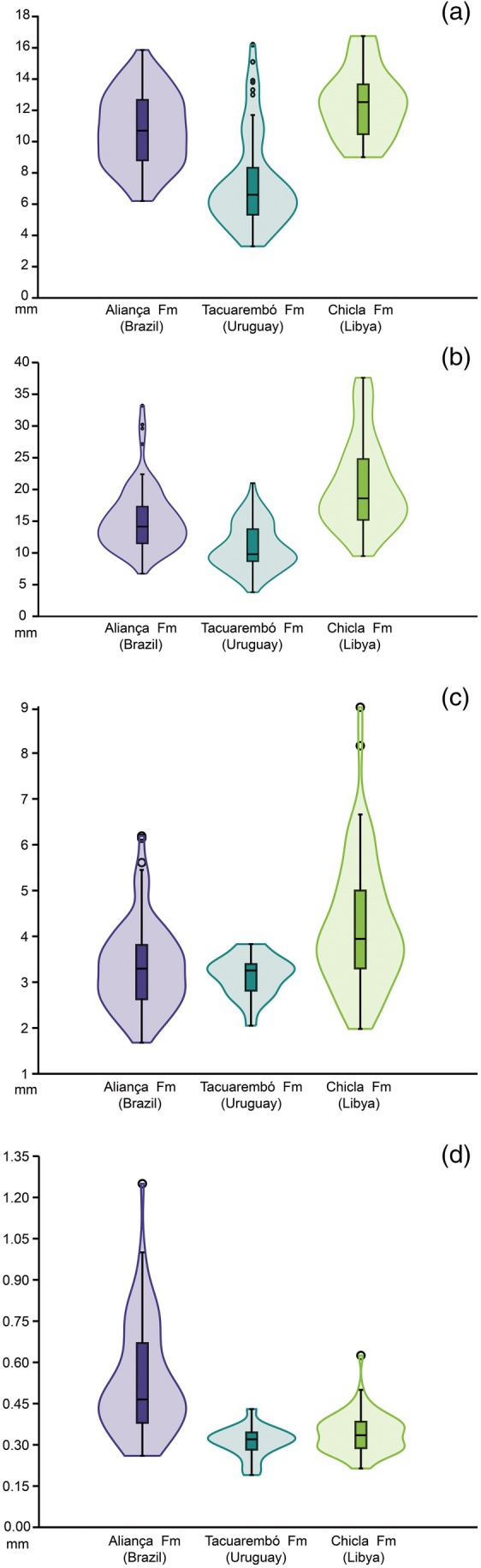
Violin boxplot graphs demonstrating the distribution of the data for tooth variation in *Priohybodus arambourgi* from different assemblages: Aliança Formation (Brazil) in purple, Tacuarembó Formation (Uruguay) in green, and Chicla Formation (Libya) in light green. (a) Crown height (HC); (b) crown length (TLC); (c) thickness at the crown base (TLC); (d) Length of a single denticle. All variables measured in millimeters, with outliers depicted by circles.

The multivariate variance analysis indicates a significant difference in the variance of tooth assemblages of *P. arambourgi* (*p*‐value lower than 0.001), demonstrating a general variation in the tooth size of *P. arambourgi* among different Gondwanan assemblages (Figure 7). However, examination of the variables shows a close resemblance or accentuated discrepancy between the assemblages, which are encompassed in Tukey's pairwise comparisons (Table 3).

**TABLE 3:** Tukey's pairwise comparison among measurements and assemblages, showing in the upper diagonal the *p*‐values; and lower diagonal the Tukey's *Q* values for relative statistical correlation. Significant comparisons are indicated in the gray boxes.

	Aliança fm. (Brazil)	Tacuarembó fm. (Uruguay)	Chicla fm. (Libya)
**HC—1**			
Aliança fm. (Brazil)		2.18E−05	0.2876
Tacuarembó fm. (Uruguay)	13.22		2.18E−05
Chicla fm.(Libya)	2131	8913	
**TLC—7**			
Aliança fm. (Brazil)		2.18E−05	2.21E−05
Tacuarembó fm. (Uruguay)	10.15		2.18E−05
Chicla fm.(Libya)	7406	16.6	
**TBC—9**			
Aliança fm. (Brazil)		0.998	2.28E−05
Tacuarembó fm. (Uruguay)	0.08589		0.008507
Chicla fm.(Libya)	7149	4193	
**DL—12**			
Aliança fm. (Brazil)		2.18E−05	2.18E−05
Tacuarembó fm. (Uruguay)	9763		0.4377
Chicla fm.(Libya)	12.11	1734	

Abbreviations: DL, length of a single denticle; HC, height of tooth crown; TBC, thickness at the base of the crown; TLC, total mesiodistal crown length.

The heights of tooth crowns from the Aliança Formation ranged from 6.2 to 15.86 mm, with a median of 10.7 mm. On the other hand, for the Chicla Formation, the heights of tooth crowns differ, ranging from 9.00 to 16.75 mm, with a median of 12.52 mm. In the Tacuarembó Formation, the same character varied from 3.30 to 16.2 mm, with a median of 6.6 mm. In general, the Chicla Formation (Libya) assemblage supports the higher medians for crown height, followed by the Aliança Formation (Brazil) and Tacuarembó Formation (Uruguay). However, there is no significant difference identified between the first two assemblages (Table 3). Indeed, the Tacuarembó Formation sample has the lower density in the distribution of higher crowns when compared with other formations, which is only observed by outliers (Figure 6a).

The mesiodistal lengths of tooth crowns from the Aliança Formation ranged from 6.73 to 33.2 mm, with a median of 14.14 mm. In the Chicla Formation assemblage, the crown lengths changed from 9.5 to 37.6 mm, with a median of 18.6 mm. In the Tacuarembó Formation assemblage, the same feature varied from 3.8 to 21 mm, with a median of 9.8 mm. The general pattern looks like that seen in the crown height. In this case, the Tacuarembó Formation has the lowest density in the distribution of longer crowns, which follows the other assemblages, with the Chicla Formation having the longest teeth (Figure 7b). However, this variable is the only one that shows significant differences among all the assemblages studied (Table 3).

The other measured variable of tooth crown size is crown thickness at the base of the crown. In the case of the Aliança Formation, this feature ranged from 1.68 to 6.19 mm, with a median of 3.295 mm. In the Chicla Formation specimens, the crown thickness ranges from 1.98 to 9 mm, with a median of 3.945 mm. In the Tacuarembó Formation, the same feature varied from 2.05 to 3.83 mm, with a median of 3.255 mm (Figure 7c). Thus, crowns in the Chicla formation assemblage are thicker than those in other assemblages (see the outliers), and the thickest teeth of all are present in this first assemblage. However, by contrast to previously considered variables in which the Chicla Formation assemblage was followed in size by the crowns from the Aliança Formation assemblages, the Aliança and Tacuarembó formations bear no significant difference in their variation (Table 3).

The measurements of single denticle length from Aliança Formation assemblage tooth crowns range from 0.26 to 1.25 mm, with a median of 0.465 mm. On the other hand, for the Chicla Formation, the denticle length ranges from 0.214 to 0.625 mm, with a median of 0.355 mm. In the Tacuarembó Formation, the same feature varied from 0.19 to 0.43 mm, with a median of 0.32 mm (Figure 7d). The Aliança Formation assemblage shows the broadest variation in size, bearing the largest denticles. In contrast to other variables, denticle size plots placed the Chicla and Tacuarembó formation assemblages as closely similar, as indicated by non‐significant variation among the assemblages (Table 3).

**FIGURE 8:**
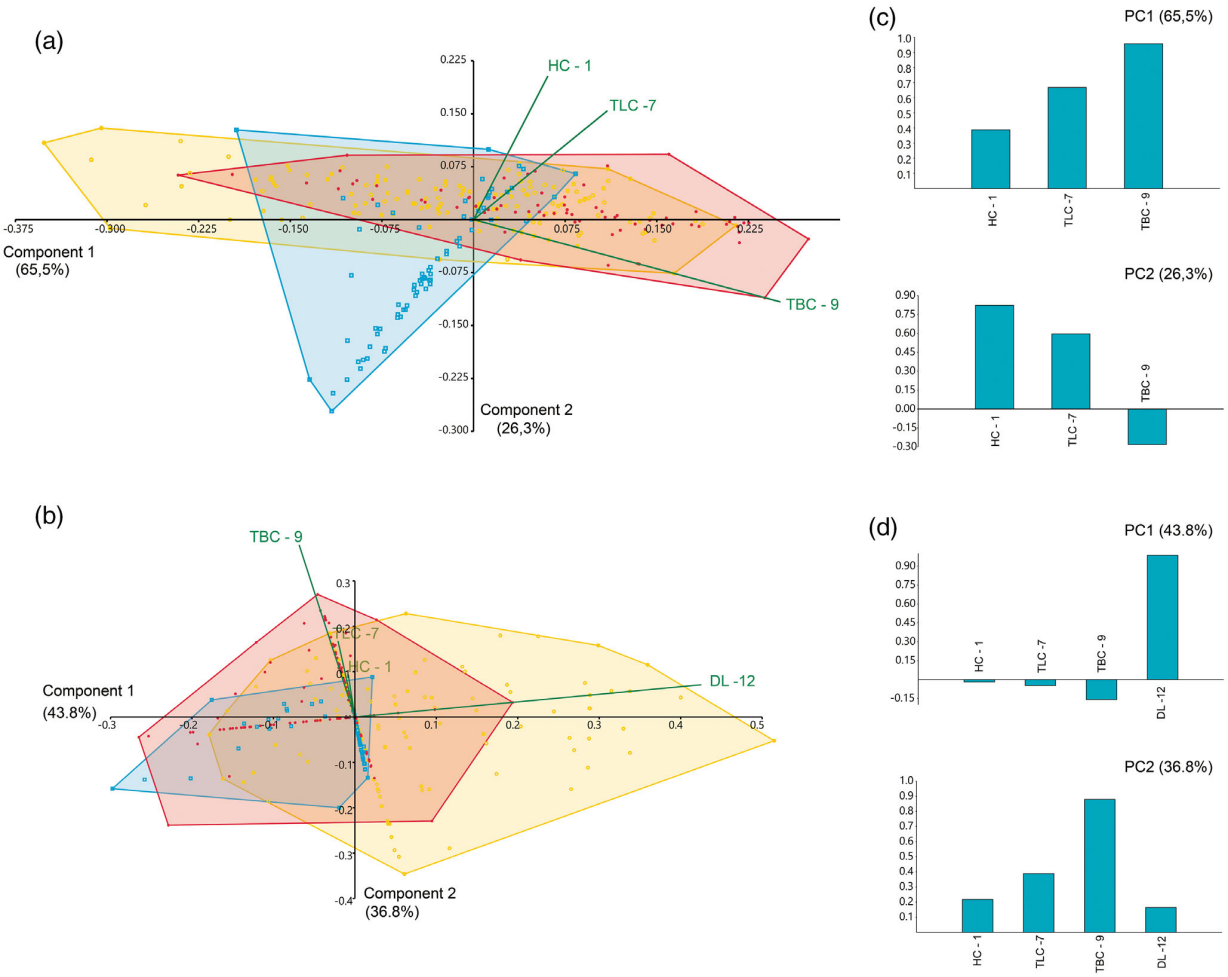
Results of principal component analysis (PCA) performed on the log‐transformed measurements represented as convex hulls for *Priohybodus arambourgi* assemblages from the Aliança Formation (yellow), Chicla Formation (red) and Tacuarembó Formation (blue); (a) Morphospace plotted on PC1 and PC2 without the feature of denticle length (DL—12), considering only height (HC—1), length (TLC—7) and thickness (TBC—9); (b) morphospace plotted on PC1 and PC2 without the feature of denticle length (DL—12); (c, d) Loading values showing the variables associated with the first two principal component axes for the first and second PCA, respectively. For measurements abbreviation see Figure 2.

The PCA compares the effect of the presence or absence of denticle length among the other size variables of the teeth, such as height, length, and thickness (Figure 8). The exclusion of the denticle variable in the first PCA results in PC1 having 65.5% of variation representation in the sample, mainly related to the thickness (TBC—9 corresponds to 0.89 of the component variation). As demonstrated by the Tukey's pairwise comparisons (Table 3), an approximation results in the morphospace occupied by the Aliança and Tacuarembó assemblages (Figure 8a). On PC2 (26.3% of the variance), crown height is the most important variable to explain the variation (Figure 8c). The morphospace occupied by the assemblages from the Chicla and Aliança Formations is recovered as well, overlain, corroborating the weak difference of the variance observed in the Tukey's pairwise comparisons. As a consequence, the data for the Tacuarembó assemblage shows significant overlapping in the lower left quadrant, reflecting the small size of the teeth from this assemblage (Table 3). Adding denticle length to the second PCA (Figure 8b) results in the PC1 corresponding to 43.8% of the variance, which is most influenced by denticle length (DL—12). As previously seen in the Tukey's pairwise comparisons, in which Tacuarembó and Chicla formation teeth bear non‐significant differences, the result is a wide superposition in their morphospaces. As the denticle length corresponds to 98% of the variation of PC1, the morphospace of the Aliança Formation assemblage is further displaced strongly to positive values of PC1 compared with the first PCA. The PC2 of the second PCA corresponds to 36.5% of the variance, corroborating the values previously observed in PC1 of the first PCA, with strong influence in the axis of the crown thickness (0.88—see Figure 8d). The DL is weakly correlated with another measurement (see Table 2), but clearly impacts the tooth morphology of the hybodontiform, *P. arambourgi*, comparison among different assemblages.

A comparison of the biplot regressions of size measurements, such as height or length of the crown, with denticle length indicates differences in their statistical significance. The Aliança Formation has no significant correlation between these variables, bearing a weak negative inclination axis between denticle length and crown height or length. This means that denticle size does not vary with the size of the crown. On the other hand, comparing with other assemblages, the biplot correlation is significant between denticle size and crown size (*p* < 0.001; *r* ^2^ = 0.47 for height; *r* ^2^ = 0.23 for length; and *r* ^2^ = 0.30 for thickness), indicating that denticle size increases proportionally with an increase in crown size. This explains why the Tacuarembó and Chicla formations have a clear approximation in Tukey's comparison and PCA morphospace.

### 3.7 Comparisons of *P. arambourgi* with modern sharks (Lamniformes)

**FIGURE 9:**
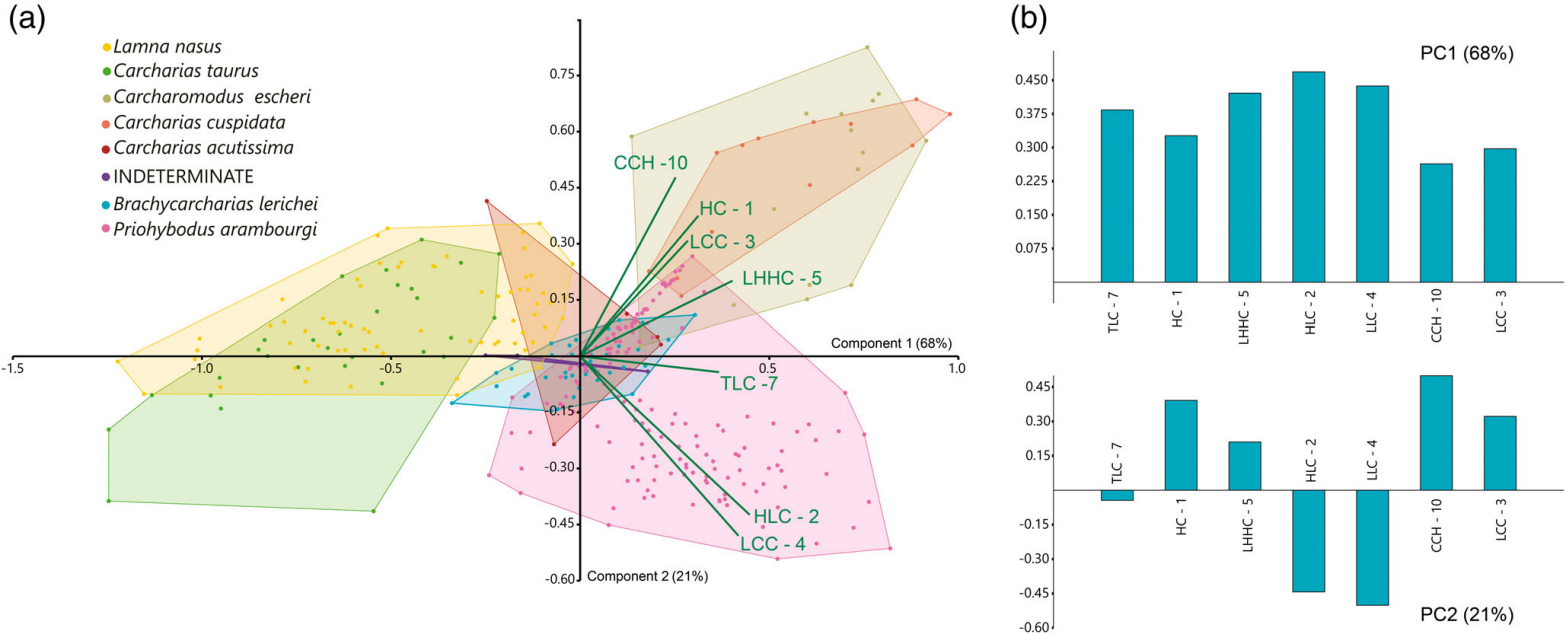
Results of principal component analysis (PCA) performed on the log‐transformed measurements represented as convex hulls for the *Priohybodus arambourgi* assemblage from the Aliança Formation (pink); (a) Morphospace plotted on PC1 and PC2, considering the Priohybodus arambourgi in the context of lamniformes from analyses of Marramà and Kriwet (2017); (b) loading values showing the variables associated with the first two principal component axes for the first and second PCA, respectively. For measurement abbreviations see Figure 2.

Some characters allow comparison in the PCA with the morphospace occupied by extant and extinct lamniform sharks. These measurements encompass the sizes of the central cusp and the lateral cusplets. The PC1 has the representation in this analysis of 68% (Figure 9). This component has positive values varying from 0.26 to 0.46, in which the values of height and length of the lateral cusplets (HLC—2 and LLC—4, respectively), as well as the length at the half‐height of the central cusp (LHHC—5), are the most significant values. The lower values in this PC1 link with the central cusp measurements (LCC—3 and CCH—10). In the first component, the variation among the characters lacks wide discrepancy, demonstrating a likely distribution of the measurements in the biplot of the first axis (Figure 9a). The morphospace of species such as *P. arambourgi*, *Carcharias cuspidata*, and *Carcharomodus escheri* appears positively related to this axis, while species such as *Carcharias taurus* and *Lamna nasus* occupy an opposite position. The second relevant component (PC2) explains 21% of the variation in the sample, reaching loading values varying from −0.50 to 0.49. Among the positive and most important measurements are those associated with central cusp height (CCH—10 and CH—1) and length (LCC—3). The negative values are related to lateral cusplet height and length (HCL—2 and LCC—4). In this component, the morphospace plotted on PC2 has *P. arambourgi* and *C. taurus* occupying negative quadrants, while most of the morphospaces of *L. nasus*, *C. escheri*, and *C. cuspidata* are plotted in positive quadrants. Taxa such as *Brachycarcharias lerichei* and *Carcharias acutissima* have a morphospace near the intersection of the components.

The distribution of different lamniformes in the morphospace of the PCA demonstrates a partial overlap compared with that of *P. arambourgi*. The overlapping is observed with *B. lerichei* in addition to weak overlapping with *C. acutissima* and *C. escheri*. This seems to be due to the measurements of the lateral cusplets. The taxon that most overlaps with the morphospace of *P. arambourgi* is *B. lerichei*, which has some lateral cusplet projection in comparison with other lamniformes. The group as a whole demonstrates an evident reduction in this feature, with the central cusp being responsible for most of the morphology of lamniformes compared herein. Thus, the overlap with *C. escheri* is mainly due to its central cusp being triangular, bearing a similar shape to that of *P. arambourgi*.

## 4 DISCUSSION

### 4.1 Implications of a new record

The new record of *P. arambourgi* in the Aliança Formation represents the first report of a hybodontiform shark in the Tucano Basin, as well as the first report of *P. arambourgi* in Brazil. A Brazilian occurrence of the species was predicted by Soto et al. (2012), taking into account the currently known distribution of the species.

Many Mesozoic fossil‐bearing deposits are known in the sedimentary basins of northeastern Brazil, whose deposition is associated with the African‐Brazilian Depression (Da Rosa & Garcia, 2000; Küchle et al., 2011; Netto & Oliveira, 1985). Among these deposits, the world‐famous Crato and Romualdo Formations (Aptian) in the Santana Group of the Araripe Basin provide a significant paleobiological window into the Mesozoic of South America (Maisey, 1991; Ribeiro et al., 2021; Varejão et al., 2019). Despite our knowledge of the Lower Cretaceous of this basin, the Jurassic record is still sparse in the Mesozoic basins of northeastern Brazil, being limited to Upper Jurassic successions, such as those of the Aliança, Bananeiras, and Brejo Santo formations (respectively in RTJ, Sergipe‐Alagoas, and Araripe basins). These are important repositories of unexplored fossil occurrences in sediments associated with the African‐Brazilian Depression (Souto & Fernandes, 2017).

Regarding the record of hybodontiforms in the aforementioned Mesozoic basins of the northeastern Brazilian margin, Brito and Ferreira (1989) described *Tribodus limae* from the Santana Formation. Hybodontiform remains (Hybodontidae and Lonchidiidae) have also been described from the Missão Velha Formation (?Lower Cretaceous) of the Araripe Basin (Cupello et al., 2012). Other hybodontiform shark remains, such as teeth, cephalic spines, dorsal fin spines, and dermal denticles, are widely distributed in the interior basins of northeastern Brazil (e.g., Sanfranciscana, Iguatú, Rio do Peixe, and Lima Campos) and have generally been referred to the genera *Hybodus* and *Polyacrodus* and associated with Hybodontiformes indet. (Brito et al., 1994; Fragoso et al., 2019). In the Malhada Vermelha Formation (Early Cretaceous) of the Lima Campos Basin, a new species of Planohybodus, *P. marki*, was proposed for this formation (Pinheiro et al., 2013; for further discussion see Stumpf, Etches, et al., 2021a).

The palaeobiogeographical significance of the new record of *P. arambourgi* presented here comprises the expansion of geographical occurrence of this species in the Late Jurassic of Gondwana, along with the Tacuarembó Formation in Uruguay and other Western Gondwanan localities, such as has already been seen in Ethiopia, Somalia, and North Yemen (Table 1). *P. arambourgi* records occur along the coastal borders of Gondwana. Since the Tucano Basin represents an intracontinental rift associated with the rupture of West Gondwana during the formation of the eastern Brazilian margin (Alvarez & Holz, 2022; Heine et al., 2013; Milani et al., 1988; Moulin et al., 2010; Szatmari et al., 1985; Szatmari & Milani, 1999), the new records from the Aliança Formation also represent an occurrence in the interior of Gondwana.

*P. arambourgi* has been regarded as a freshwater shark in North Africa deposits (Anderson et al., 2007; Cuny et al., 2004), as well as in the Tacuarembó Formation, Uruguay (Soto et al., 2012). In the Aliança Formation, the deposits represent the same palaeoenvironmental conditions as those of the Tucano Basin. The stratigraphic pre‐rift sequence (Brotas Group) is marked by recurrence of fluvial‐eolian cycles (Sergi formation) and lacustrine transgressions (Aliança Formation). The phase of continental, shallow, and perennial lakes with fluvio‐deltaic influence represented by the Aliança Formation is confirmed by the presence of lacustrine ostracods and palynological records (Brito, 1987; Guzmán‐González et al., 2016; Viana et al., 1971). Aliança Formation crops out in the RTJ basins, and especially in the Jatobá Basin, commonly preserves vertebrate remains that include those of other hybodontiform sharks, *Mawsonia*, Dipnoi, and other fishes, crocodylomorphs, and dinosaur remains. Addressing the Aliança Formation in the Tucano Basin, the occurrence of pteridophytes associated with this recently discovered paleofauna confirms a freshwater environment for *Priohybodus*.

The stratigraphic range of *P. arambourgi* had been considered to reach the Lower Cretaceous Aptian/Albian (Cuny et al., 2004; Duffin, 2001), although the Tunisian Douiret Formation and its Libyan equivalent were later regarded as pre‐Aptian, and probably Hauterivian/Barremian in age (Cuny et al., 2010; Le Loeuff et al., 2010). *P. arambourgi* remains occur at the base of the Tacuarembó Formation, which is regarded as Late Jurassic in age due to the joint occurrence of the theropods *Ceratosaurus* and *Torvosaurus* and the presence of a *Gnathosaurus*‐like pterosaur (Soto et al., 2020a, 2020b, 2022). Based on biostratigraphical evidence, such as lacustrine ostracods and palynological records, the Aliança Formation was most likely deposited during the Tithonian (Almeida‐Lima et al., 2022; Da Rosa & Garcia, 2000; Guzmán‐González et al., 2016; Müller, 1966; Regali et al., 1974; Regali & Viana, 1989; Viana et al., 1971). Thus, the record of *P. arambourgi* in the Aliança Formation is in concordance with the previously proposed biochron of the genus, encompassing Tithonian/Early Cretaceous strata (Soto et al., 2012).

**FIGURE 10:**
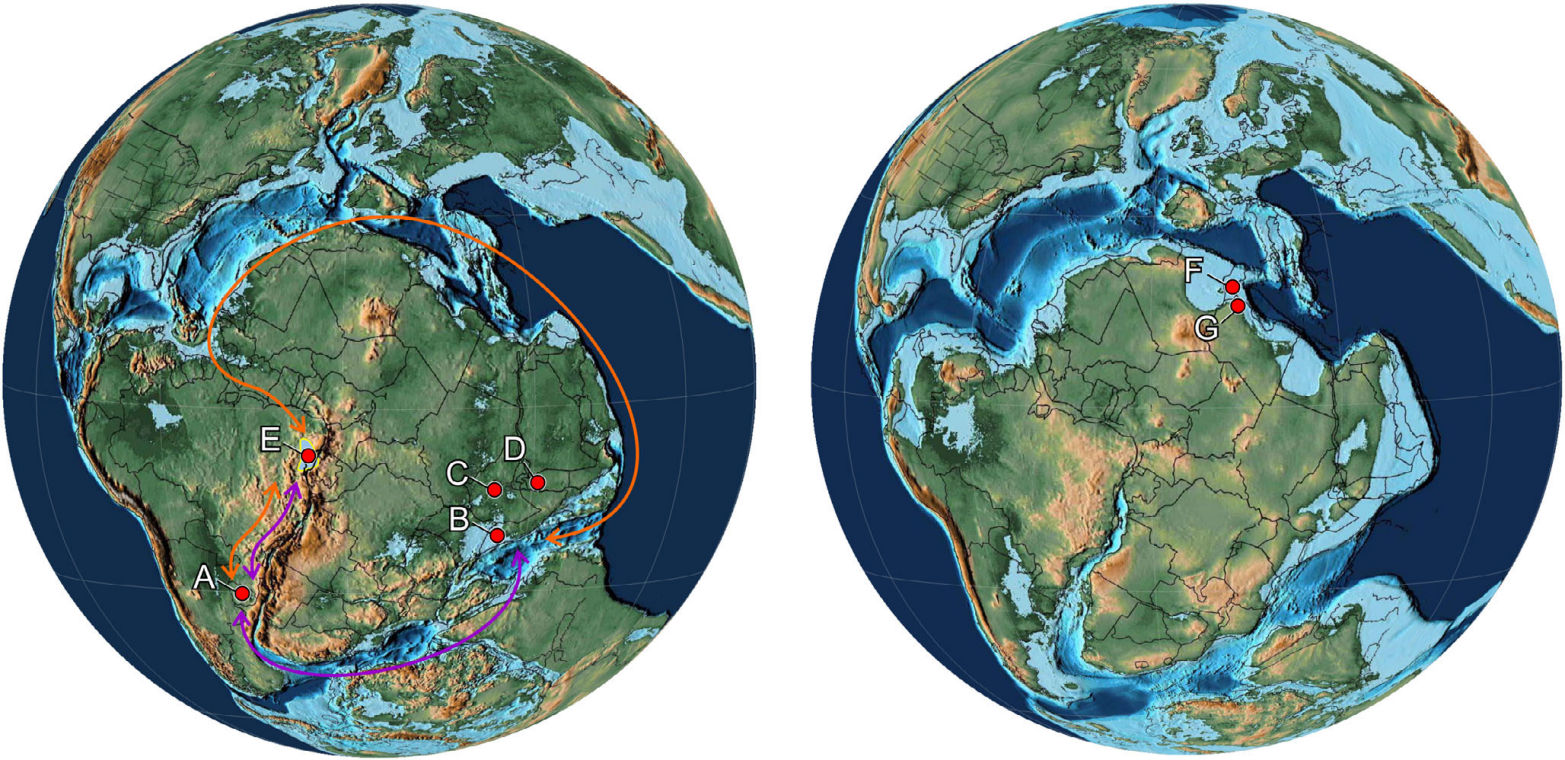
Geographical distribution of *Priohybodus arambourgi* during the Tithonian (Jurassic) (left) and Barremian (Cretaceous) (right); (a) Tacuarembó Formation, Uruguay; (b) Lugh Series, Somalia; (c) Mugher Mudstone, Ethiopia; (d) Amran Formation, North Yemen; (e) Aliança Formation, Brazil; (f) Chicla Formation, Libya; (g) Douiret Formation, Tunisia. With possibly South American or Eastern African origins in Jurassic, a coast dispersal route through Northern Gondwana (orange arrow) or Southern Gondwana (purple arrow) based on coastal. Paleomaps were modified from Scotese (2016).

*P. arambourgi* belongs, in the Hybodontidae (Cuny et al., 2003, 2004; Duffin, 2001), to a subclade whose members bear specialized high‐crowned, multicuspid teeth (Hybodontinae sensu Maisey, 1989). Alternatively, the taxon has been placed in an unnamed subfamily, along with *Planohybodus* and *Secarodus*, a sister group of the Hybodontinae that is restricted to *Hybodus* and *Egertonodus* (Rees, 2008). The North African occurrences of *P. arambourgi* are younger (Early Cretaceous of Tunisia and Libya) compared with the records from Southern and Eastern Gondwana. This suggests that the species migrated from the south northwards from Jurassic to Cretaceous times (Soto et al., 2012). However, the Hybodontidae has a European record and consequently a northern ancestry, as *Secarodus* has been recorded in the Bathonian, Middle Jurassic, of southern England. The genus *Planohybodus* has been recorded in the Early Jurassic of Luxembourg (Delsate, 1995; Rees & Underwood, 2008) and the Middle Jurassic (Bathonian) of France, Scotland, and England (Rees & Underwood, 2008). Late Jurassic remains occur in northeastern Spain, France, and England (Bermúdez‐Rochas et al., 2009; Underwood, 2002; Vullo, 2011). This suggests that the clade containing *Secarodus*, *Planohybodus*, and *Priohybodus* had emerged in Europe during the Jurassic, implying an endemic distribution of this group in marine paleoenvironments of Europe during the Jurassic period, and later geographical range expansion to Gondwana during the later Mesozoic. However, this suggestion is contradicted by the stratigraphical and geographical occurrences of *P. arambourgi* in South America during the Late Jurassic, as well as the record of *Planohybodus* in the basins of northeastern Brazil (Cupello et al., 2012; Pinheiro et al., 2013) and Asia (Sharma & Singh, 2021). Two points deserve attention: the first is the lack of a phylogenetic framework supported by numerical cladistic analyses for the hybodontiforms, which limits any further understanding of the origins and relationships of the clade. Second, the lack of extensive records regarding the Mesozoic hybodontiform fauna of South America hinders tracing the history of the clade due to the lack of both material and documentation (Cupello et al., 2012). In parallel, in the context of the RTJ, the collection of more than 500 isolated teeth of hybodontiforms from the Aliança Formation in the Jatobá Basin, none of which are attributed to *P. arambourgi* (França et al., 2019), indicates that *P. arambourgi* was restricted to the Tucano Basin, and may represent an ecological regionalization of the Aliança Formation during the Jurassic, or even that a geographical barrier separated the northern and southern portions of the RTJ basins. Therefore, the northern examples (Jatobá Basin) would have had more contact with Laurasian faunas, while the southern portion (Recôncavo—Tucano Basins) was more influenced by Gondwanan faunas (Figure 10, purple arrow).

### 4.2 Uniqueness of dental morphology

*P. arambourgi* demonstrates a strong correlation among morphometric features found in the crown size. These include the height, mesiodistal length, and crown thickness, showing that, in *P. arambourgi*, the teeth increase proportionally in size (Table 2, Figure 5). Significant differences in the sizes of *P. arambourgi* teeth from different assemblages demonstrate that variation occurred between different Gondwanan populations of the species (Table 3, Figure 7). In general, the teeth from the Chicla Formation are the larger ones, followed by those from the Aliança Formation assemblage. The smallest teeth are those from the Tacuarembó formation assemblage (Figure 8a). The morphology of *P. arambourgi* is clearly uniform, considering the morphology of teeth from different assemblages. It allows for a confident identification of the species based on its initial diagnosis by Duffin (2001). However, variation among different populations would be expected as a consequence of the wider geographical occurrences of this hybodontiform. Perhaps the strongest example of such variation in allopatric populations is the presence of ornamentation on the central cusp of the Tunisian specimen together with the possession of more upright lateral cusplets in comparison with other occurrences (Cuny et al., 2004).

The high‐crowned, relatively homodont dentition suggests that *P*. *arambourgi* shares a cutting‐dentition type with extinct and extant neoselachians (Cappetta, 1986; Duffin, 2001). Both neoselachian sharks with well‐developed cutting‐type dentitions, such as lamniformes and carcharhiniforms, exhibit an evident heterodonty in their dentition (Cappetta, 2012; Cullen & Marshall, 2019). *P. arambourgi* represents a shark‐like hybodontiform with remarkable cutting‐type dentition, but is a bearer of evident homodonty, mainly compared with that observed in *Hybodus* and other hybodontiforms. Although having a rather homodont dentition, the teeth of *P. arambourgi* show slight asymmetry, revealed by the inclination of the central cusp and lateral cusplets. The more asymmetric teeth were probably lateral in position (Duffin, 2001; Perea et al., 2001). In our study, the inclination of the lateral cusplets relative to the central cusp and the curvature on the lingual surface of the central cusps demonstrate that the dentition of *P. arambourgi* is rather homodont, and that the teeth only changed morphometrically in crown size. Our data corroborates the homodonty of *P. arambourgi* originally observed by Duffin (2001), Perea et al. (2001), and Soto et al. (2012), and demonstrates that homodonty is not associated with any quantitative character, varying homogeneously in the sample. On the other hand, in the dentitions of other high‐crowned hybodontiforms, such as *Secarodus*, disjunct monognathic heterodonty is present, with anterior teeth being almost symmetrical in profile, resulting in a similar pattern of heterodonty to that of *Durnonovariaodus* (Stumpf, López‐Romero, et al., 2021).

The wear area variation in the sample is not linked proportionally with crown size variables. It may be considered as a potential ecological trait, in which the variation could be related to feeding mechanisms and prey preferences, rather than representing a morphometric development of the teeth. In general, this area is represented by a concave notch on the apex of the central cusp, giving the impression of breakage. The feature is observed in a number of specimens of *P. arambourgi* from the Aliança Formation (Figure 3g,h: specimen LAPAL/SBF‐2‐005; Figure 3o,p: specimen LAPAL/SBF‐2‐022; Figure 4: specimens LAPAL/SBF‐2‐0164 and LAPAL/SBF‐2‐0186), as well as other specimens, such as those from Libya (Duffin, 2001—Figure 1: specimen NHMUK PV P65466, NHMUK PV P65467) and Uruguay (Perea et al., 2001—plate 1: specimen FC‐DPV‐1008; Soto et al., 2012—Figure 1: specimen FC‐DPV‐1609).

**FIGURE 11:**
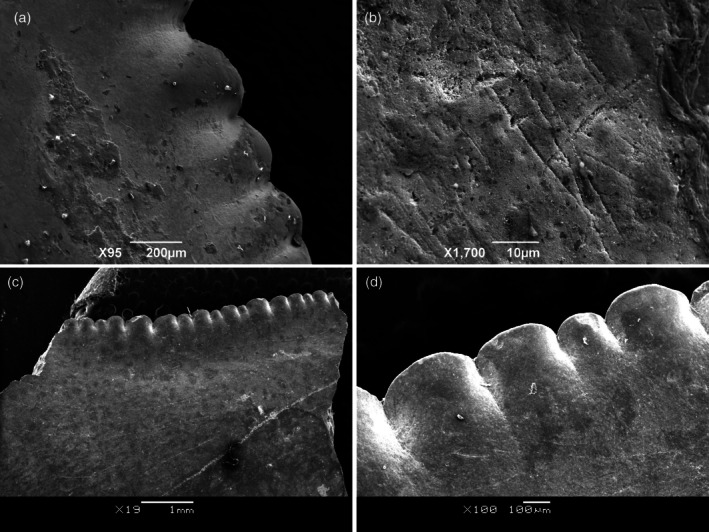
Details of denticles from the central cusp, showing the scratches on the enameloid surface of *Priohybodus arambourgi* from the Aliança Formation of Brazil (a, b) LAPAL/SBF‐2‐0229) and the Tacuarembó Formation of Uruguay (c, d, FC‐DPV 1144).

The rarity of high‐crowned serrated teeth among hybodontiforms might be explained by their lack of the triple‐layered enameloid that characterizes modern sharks (Duffin & Cuny, 2008; Gillis & Donoghue, 2007). However, even bearing a well‐developed central cusp, microstructural analysis on enameloid indicates that *P. arambourgi* has a single layer, as seen in the specimen from Tunisia (Cuny et al., 2018). It suggests that the central cusp was not very resistant to tensile stress, and the feeding ecology of the species has been inferred as comprising large, soft‐bodied prey as opposed to heavily armored semionotiform fish (Cuny et al., 2008; Duffin, 2001; Perea et al., 2001; Soto et al., 2012). In addition to the morphological evidence of the wear surface on the central cusp, the enameloid surface also shows a number of scratches on the enameloid, observed in Scanning Electron Microscopy micrographs of central cusp denticles (Figure 11). Despite the crowns apparently being adapted for cutting up soft‐bodied prey, the feeding ecology of *P. arambourgi* might have resulted in damage and abrasion on the enameloid by occasional opportunistic preying upon harder‐bodied fishes, such as members of the Semionotiformes, which are very abundant in the fossil‐bearing sites of the Tacuarembó and Aliança Formations (Eltink et al., 2018; Perea et al., 2001). In modern sharks with durophagous feeding habits, such as the Port Jackson shark, *Heterodontus portusjacksoni*, wear facets are present on the top of the central cusp; this species has developed a central cusp composed entirely of enameloid, without any dentine penetrating the central cusp, contrary to the situation in other modern sharks (Moyer & Bemis, 2017). As a result, the erosion is limited to the enameloid layer, and the underlying dentine is never exposed to contact (Amini et al., 2020). In the case of *Priohybodus*, harder prey items processed by teeth having a single crystallite enameloid layer and possibly pseudosteodont histology might result in a pattern of wear erosion in which the dentine layer becomes exposed (the worn area). Further investigation of tooth histology in *P. arambourgi* is needed to unveil the enameloid microstructure in this hybodontiform (Cuny et al., 2018).

Hybodontiforms with serrations on their teeth are relatively uncommon in the evolutionary context of the group, occurring several times independently in the evolution of hybodonts (Cuny et al., 2008). As seen in *Priohybodus*, forms such as *Pororhiza molimbaensis* from the Albian of the Congo (Casier, 1969), *Thaiodus ruchae* from the Aptian–Albian of Thailand, Tibet, and China (Cappetta et al., 1990, 2006; Cuny et al., 2008; Mo et al., 2015), and *Mukdahanodus trisivakulii* from the pre‐Aptian of Thailand (Cuny et al., 2009; Teng et al., 2019) are hybodontiforms with serrations along the cutting edges of the teeth. In spite of the resemblance in their serrations, these taxa differ from each other in details of crown morphology, in which the central cusp is either absent, very low, or blunt. The occurrences of *P. molimbaensis*, *T. ruchae*, and *M. trisivakulii* are restricted to the Lower Cretaceous. In this context, *P. arambourgi* had the earliest fully serrated dentition, appearing earlier than the other serrated taxa, in the Late Jurassic (Duffin, 2001). *T. ruchae* and *M. trisivakulii* are endemic to Southern Asia (Cuny et al., 2008), and *P. molimbaensis* to Central Africa (Cappetta, 1987), while *P. arambourgi* has a much wider geographical distribution, extending around Gondwana. It demonstrates that cutting feeding strategies based on fully serrated teeth, which are seen in hybodontiforms confined to specialized niches in freshwater environments (Cuny et al., 2003, 2004, 2009; Rees, 1998; Reif, 1988), are also found a wider distribution, especially in Gondwana during the middle of the Mesozoic. The appearance of cutting dentitions occurred in numerous chondrichthyan lineages, including hybodontiforms (see above), neoselachians (e.g., carcharhiniforms and lamniformes) and problematic forms of uncertain phylogenetic relationships (e.g., *Carcharopsis*) (Cappetta, 1986; Duffin, 2001; Duffin & Cuny, 2008).

The pattern of denticles making up the serrations also varies among serrated tooth hybodontiforms. In *M. trisivakulii*, the denticles are rather irregular and complex, with each denticle divided at its apex by a short groove. In apical view, some of the crowns are slightly arched lingually (Cuny et al., 2009). The species also exhibits a faint sulcus between the denticles. In *T. ruchae*, the denticles have pronounced asymmetry regarding their sizes, with some of them along the basal cutting edge of the central cusp being somewhat similar to the extant Tiger Shark, *Galeocerdo cuvier*, which exhibits secondary denticles on the distal heel of the teeth. *T. ruchae* also exhibits deeper sulci between the denticles and the absence of arching of the denticles in apical view. In *P. arambourgi* the pattern is somewhat similar to *M. trisivakulii* and *T. ruchae* in that each denticle bears a rounded crest, especially on the central cusps (Figure 3c,d). However, in *P. arambourgi* the denticles are more regular in size compared to those in *T. ruchae*. *P. arambourgi* also shows smooth variation among the different populations of assemblages from Africa and South America. The denticles of the specimens from Libya have a more square‐shaped outline, with smooth divisions in some of the denticles, somewhat resembling those of *M. trisivakulii*. On the crest of the denticles, along the line of the cutting edge, *P. arambourgi* also shows a gentle enameloid ridge, which is comparable to the situation in the denticles of the modern Great White Shark, *Carcharodon carcharias*, in contrast to the denticles (primaries and secondaries) found in the Tiger Shark, *G. cuvier*, or the asymmetrical denticles of the Blue Shark, *Prionace glauca* (Moyer & Bemis, 2017).

According to Cuny et al. (2004), in the description of *P. arambourgi* from Tunisia, between 3 and 4 serrations are present per mm. This corresponds to denticle lengths varying from 0.3 to 0.25 mm. The average values for denticle length in specimens from the Tacuarembó Formation are 0.355 mm, and 0.32 mm for the Chicla formation. This data differs from that previously reported for *P. arambourgi* teeth from Libya, which suggested there were 12 serrations for each 5 mm, corresponding to 0.4 mm per denticle (Duffin, 2001). In this context, the denticle lengths for the Aliança assemblage represent the largest single average denticle size, with a median of 0.465 mm, and the broadest size variation in comparison with those from the Chicla and Tacuarembó formations. The denticles in the latter assemblages are quite similar in size. This is indicated by non‐significant variation among the latter assemblages (see Table 2 and results of PCA in the Figure 8). The DL is weakly correlated with other measurements of the crown size (see Table 2), but clearly impacts the tooth morphology of *P. arambourgi* when comparing different assemblages.

A comparison of the biplot regressions of size measurements, such as crown height or length, with denticle length indicates no significant correlation in the Aliança Formation assemblage. However, comparing these data with that derived from other assemblages, the biplot correlation is significant between denticle size and crown size (height, length, and thickness) (see Section 3). The denser serration in the smaller teeth of *P. arambourgi* from Tunisia was noted by Cuny et al. (2004). Our results demonstrate a partial corroboration of this hypothesis, especially for the Chicla and Tacuarembó assemblages, indicating that as the crown gets larger, so the denticle size increases, and smaller crowns have increasing denticle density. This explains why the Tacuarembó and Chicla formation teeth have a clear approximation in the Tukey's comparison and PCA morphospace. However, this is not observed in the Aliança Formation assemblage. The maintenance of identical denticle length for crowns in the Aliança Formation assemblage, as in the crowns from the Tacuarembó and Chicla formations, demonstrates a perceptible variation in size, which could be the result of an unidentified but increasing directional selection pressure exerted on geographically separated populations (Cuny et al., 2009).

### 4.3 Comparison with modern sharks and paleoecological inferences

According to Cooper et al. (2023), tooth size (crown height and width) in modern sharks was found to be a reliable proxy for ecological traits (body size, prey preference, and feeding mechanism). For instance, tooth height has been widely used as an indicator of body size in lamniform sharks (e.g., Condamine et al., 2019; Shimada et al., 2020). Linear regression analyses indicate that tooth size traits (length and height) are strongly linearly correlated for the estimation of body size in sharks (Cooper et al., 2023). Applying the Cooper et al. (2023) classification to tooth lengths for different assemblages of *P. arambourgi*, we found a categorization for those from the Aliança and Tacuarembó formations as slender and wide (with the latter having the smallest ones), while teeth from the Chicla Formation are best classified as wide in the classification. This variable was the only one to show significant differences among all the assemblages sampled in this study (Table 3). Considering the height (HC) and length (TLC) of the teeth, the *P. arambourgi* populations of both the Aliança and Chicla formations were classified as medium‐sized (see Table 3), with the Tacuarembó formation assemblage classified as medium‐size as well, but being the only assemblage also to encompass small‐sized. As a consequence, the body size estimation for *P. arambourgi* would be inferred in general as being of medium size, reaching from 2 to 4 m. Previous estimations of body size for *P. arambourgi* range from 1.5 m (Perea et al., 2001) to more than 2 m (Cuny et al., 2004). However, the particular tooth size variation in specimens from the Tacuarembó Formation allows us to suggest a smaller body size for this particular population, reaching up to 2 m. Our interpretation, being based on a large number of dental morphometric dimensions, supports the previous estimation for body size in *Priohybodus* but demonstrates variation among different populations of *P. arambourgi*.

A comparison of body size with approximately coeval hybodontiforms, such as *Durnonovariaodus*, reveals a similar estimation for body size; the species also reaches a maximum length of up to 2 m (Stumpf, López‐Romero, et al., 2021). In this case, size estimation was based on more complete skeletal material (Urlichs et al., 1994). It is not uncommon for some hybodontiforms to exceed an estimated maximum body size of up to 3 m in length, especially in those taxa that are known to have inhabited open marine environments, such as *Asteracanthus*, *Planohybodus*, and *Strophodus* (e.g., Citton et al., 2019; Leuzinger et al., 2015, 2017; Stumpf, Etches, et al., 2021a; Szabó & Főzy, 2020; Underwood, 2002). However, hybodontiforms became less abundant in marine environments as neoselachian lineages underwent adaptive radiation. In the Jurassic, hybodontiforms were gradually displaced from marine environments to specialized niches (Cuny et al., 2003, 2004, 2009; Rees, 1998; Reif, 1988). Compared with those marine forms, *P. arambourgi* still bears cutting dentition in freshwater environments, representing a large Jurassic/Cretaceous hybodontiform.

Comparing the teeth of *P. arambourgi* with those of certain lamniformes, the overlapping observed with such taxa as *B. lerichei*, and the rather weaker overlap with *C. acutissima* and *C. escheri*, demonstrates the tooth morphology in terms of the lateral cusplets (Figure 8). The teeth of *B. lerichei* develop some lateral cusplet projection in contrast to other lamniformes (Marramà et al., 2018). The lamniformes demonstrate a reduction in lateral cusplet projection, with the central cusp being responsible for the greater part of the crown structure. The overlap with *C. escheri* is mainly due to its central cusp being triangular. The species has more pointed and narrow lower teeth for grasping prey and broader blade‐like upper teeth for cutting (Kriwet et al., 2015). The comparison with lamniformes indicates how peculiar the teeth of *P. arambourgi* are, sharing high crown height, which may be associated with grasping and cutting strategies during feeding, combined with a multicuspidate crown. In extant neoselachians, a tooth morphology associated with well‐developed lateral cusplets is found in the Hexanchidae, such as the Sixgill Shark, *Hexanchus griseus*. This group differs from *P. arambourgi* due to the fact that the lateral cusplets are distally oriented in the lower jaw, providing an effective cutting edge for feeding. The upper jaw teeth in *Hexanchus* have fewer cusps and are more pointed, resulting in a grasping mechanism (Whitenack & Motta, 2010). Another modern shark with multicuspidate teeth is the orectolobiform Nurse Shark, *Ginglymostoma cirratum*. This species has teeth well designed for grabbing and gripping hard‐bodied prey, as well as breaking through hard shells and exoskeletons. Sharks with clutching‐type dentitions feed close to the ocean floor and usually live inshore to hunt their low‐lying prey (Motta et al., 2008). Further multicuspidate teeth are found in the Heterodontiformes, such as the Horn Shark, *Heterodontus francisci*. The anterior teeth are small and pointed, sporting a central cusp flanked by a pair of lateral cusplets. Lateral teeth are larger and wider; their molariform structure is used for crushing (Compagno, 2002; Ebert, 2003).

A robust correlation between tooth morphology and prey preference is expected given that the primary function of teeth is to capture and process prey (Cappetta, 2012). The presence of serrations on the cutting edge has been used to infer prey preference and feeding mechanism (Ciampaglio et al., 2005; Kent, 1994). According to Cooper et al. (2023), the type of cutting edge, crown height, and length were by far the most common proxies for prey preference. Analyzing crown height, *P. arambourgi* would have had a preference for fishes and large vertebrates, based on the medium height of the tooth crowns. However, the Tacuarembó assemblage also encompasses the smaller teeth, suggesting a prey preference that also included invertebrates. In terms of crown length, *P. arambourgi* would be categorized, in general, as slender to wide. In consequence, the prey preference is likely to have included items such as fishes and larger invertebrates. The presence of serrated cutting edges, so well marked in *Priohybodus*, reinforces this suggestion. In summary, the slight differences found among the different assemblages of *P. arambourgi* might also indicate different prey preferences, which, together with variation in estimated body size, lead to the suggestion that, paleoecologically, *P. arambourgi* occupied several different niches at different times and in different places.

Extant freshwater sharks are very uncommon, but a consideration of the diets of representative taxa, such as the River Sharks (Carcharhinidae), *Glyphis garricki* or *Glyphis glyphis*, is informative. *Glyphis* has a heterodont dentition consisting of wide, triangular, serrated teeth in the upper jaw and slender and tall teeth, with faint secondary cusplets on the lower jaw (Compagno et al., 2008). Dietary data collected for *Glyphis* inhabiting Australian rivers support the contention that these animals are morphologically adapted to finding prey items in turbid, benthic‐associated environments (Peverell et al., 2006). The presence of benthic crustaceans, burrowing gobies and gudgeons, and the bottom‐feeding jewelfish and boney bream in the stomachs of *Glyphis* from the Wenlock River indicates that they prey on items taken from such habitats (Peverell et al., 2006). In addition to bony fishes, stingrays are likely to be important dietary items for adult *G. glyphis* (White et al., 2015). Another freshwater selachian, the Bull Shark, *Carcharhinus leucas* (Carcharhinidae) is encountered in rivers and estuaries (Compagno, 2002). The diet of the Bull Shark consists almost exclusively of fishes, such as ariid catfishes and stingrays, but crustaceans also have been found in their stomachs (Snelson Jr et al., 1984). The heterodont dentition of *C. leucas* comprises teeth with a wide triangular central cusp, with serration along the cutting edge. In these aforementioned cases, the prey preference based on dental morphology infers it as being composed exclusively of fishes and large vertebrates. However, in freshwater environments, opportunistic feeding ecology may include crustaceans and other invertebrates as food sources. The fact that hybodontiforms became less significant in marine environments, coincident with neoselachian radiation, leading to their being gradually displaced from marine environments to specialized niches in freshwater environments in the Jurassic (Cuny et al., 2003, 2004, 2009; Rees, 1998; Reif, 1988), would explain the presence of *P. arambourgi* as a freshwater hybodontiform shark in the Aliança Formation; it is entirely plausible that its prey preference extended beyond fishes (with hard bodies as discussed above) and occasionally large vertebrates.

## 5 CONCLUSION

In this study, we present a new record of hybodontiform *P. arambourgi* in the Aliança Formation, Tucano Basin, which represents the first description of the taxon in the Mesozoic basins of northeastern Brazil. *P. arambourgi* demonstrates proportional correlations in the increasing size of the crowns. New features presented in the tooth assemblage, such as ante‐mortem wear on the central cusp, may indicate that the feeding preference of *P. arambourgi* was harder‐bodied fishes. We corroborate the characterization of homodonty in *P. arambourgi*. The presence of a homodont dentition consisting of almost symmetrical, high‐crowned, and multicuspid teeth, in combination with prominent serrations in the cutting edge of the main cusp and lateral cusplets, makes *P. arambourgi* unique not only among hybodontiforms but also among neoselachians, extinct and extant.

A comparison among different populations from Gondwanan assemblages (Chicla Formation—Lybia; Aliança Formation—Brazil; and Tacuarembó Formation—Uruguay) containing *P. arambourgi* demonstrates some intra‐specific variation concerning the tooth size. However, denticle measurements are shown to be independent of size‐increasing characters in the teeth of *P. arambourgi*.

The tooth size in *P. arambourgi* (crown height and length) was used as a proxy to infer ecological traits of extant sharks (body size, prey preference, and feeding mechanism). Based on that, *P. arambourgi* would reach from 2 to 4 m in size. The comparison of the Gondwanan teeth with those of some fossil lamniformes indicates how peculiar the teeth of *P. arambourgi* actually are, sharing a high crown profile, but with a multicuspidate crown, which may be associated with cutting and grasping strategies for feeding. *P. arambourgi* would have as prey preferences fishes and large vertebrates, based on the height of the teeth. In sum, the slight differences found in different assemblages of *P. arambourgi* might also indicate differences in prey preferences, which, together with variation in body size, demonstrate the presence of slight differences in the niche occupations of *P. arambourgi* in the Mesozoic of Gondwana.

## AUTHOR CONTRIBUTIONS

**Estevan Eltink:** Funding acquisition; investigation; conceptualization; writing – original draft; methodology; visualization; validation; writing – review and editing; formal analysis; software; project administration; data curation; supervision; resources. **Kelly Roberta da Silva:** Investigation; validation; methodology; data curation; formal analysis; writing – original draft; software. **Marco Aurélio Gallo de França:** Conceptualization; investigation; funding acquisition; writing – review and editing; visualization; data curation; methodology. **Débora Melo Ferrer de Morais:** Writing – original draft; investigation; data curation; methodology. **Matías Soto:** Investigation; writing – review and editing; data curation; visualization; methodology. **Christopher J. Duffin:** Data curation; writing – review and editing; investigation; visualization; methodology.

## FUNDING INFORMATION

Fundação de Amparo à Ciência e Tecnologia de Pernambuco (FACEPE) to Estevan Eltink (APQ‐1119‐1.07/21); Conselho Nacional de Desenvolvimento Científico e Tecnológico (CNPq) and Fundação de Amparo à Pesquisa do Estado da Bahia (FAPESB) (0497/2023, 5171/2019) to Kelly Roberta da Silva; Jurassic Foundation grant to Matías Soto.

## Supporting information


**Data S1.** Supporting Information.

**Data S2.** Supporting Information.

**Data S3.** Supporting Information.
